# Treatment with integrase inhibitor suggests a new interpretation of HIV RNA decay curves that reveals a subset of cells with slow integration

**DOI:** 10.1371/journal.ppat.1006478

**Published:** 2017-07-05

**Authors:** E. Fabian Cardozo, Adriana Andrade, John W. Mellors, Daniel R. Kuritzkes, Alan S. Perelson, Ruy M. Ribeiro

**Affiliations:** 1 Theoretical Biology and Biophysics, Theoretical Division, Los Alamos National Laboratory, Los Alamos, NM, United States of America; 2 The Johns Hopkins University, Baltimore, MD, United States of America; 3 University of Pittsburgh School of Medicine, Pittsburgh, PA, United States of America; 4 Brigham and Women’s Hospital, Harvard Medical School, Boston, MA, United States of America; 5 Laboratório de Biomatemática, Faculdade de Medicina, Universidade de Lisboa. Av. Professor Egas Moniz, 1649–028 Lisboa, Portugal; Vaccine Research Center, UNITED STATES

## Abstract

The kinetics of HIV-1 decay under treatment depends on the class of antiretrovirals used. Mathematical models are useful to interpret the different profiles, providing quantitative information about the kinetics of virus replication and the cell populations contributing to viral decay. We modeled proviral integration in short- and long-lived infected cells to compare viral kinetics under treatment with and without the integrase inhibitor raltegravir (RAL). We fitted the model to data obtained from participants treated with RAL-containing regimes or with a four-drug regimen of protease and reverse transcriptase inhibitors. Our model explains the existence and quantifies the three phases of HIV-1 RNA decay in RAL-based regimens vs. the two phases observed in therapies without RAL. Our findings indicate that HIV-1 infection is mostly sustained by short-lived infected cells with fast integration and a short viral production period, and by long-lived infected cells with slow integration but an equally short viral production period. We propose that these cells represent activated and resting infected CD4+ T-cells, respectively, and estimate that infection of resting cells represent ~4% of productive reverse transcription events in chronic infection. RAL reveals the kinetics of proviral integration, showing that in short-lived cells the pre-integration population has a half-life of ~7 hours, whereas in long-lived cells this half-life is ~6 weeks. We also show that the efficacy of RAL can be estimated by the difference in viral load at the start of the second phase in protocols with and without RAL. Overall, we provide a mechanistic model of viral infection that parsimoniously explains the kinetics of viral load decline under multiple classes of antiretrovirals.

## Author summary

Antiretroviral therapy in HIV-1 infection leads to characteristic viral load decay profiles. Interpretation of this profile with mathematical models has provided important insights into the dynamics of the viral lifecycle (e.g., the turnover of infected cells). Here we develop a unified model and analyze viral load data from HIV-infected participants under treatment with and without an integrase inhibitor. Our model offers a new explanation for the observed differences in the decay profiles, and strongly supports the hypothesis that the second phase of viral decay is due to the loss of long-lived resting CD4+ T-cells with slow proviral integration. We estimate parameters of this decay and estimate the efficacy of an integrase inhibitor.

## Introduction

Viral dynamics analysis is a powerful tool to probe the lifecycle of viral infections [1–3]. In the case of HIV-1, treatment with reverse transcriptase inhibitors (RTI) and protease inhibitors (PI) resulted in a two-phase decline in plasma viral load [4–6]. Mathematical modeling attributed the first phase to the loss of productively infected cells with a short half-life (*t*_1/2_~0.7 days), presumably activated CD4+ T-cells [7]. The second phase was attributed to the loss of “long-lived” infected cells with a slower loss rate (*t*_1/2_ ~14 days) [4]. The nature of the cells responsible for this second phase is still debated, and possibilities include, among others, macrophages or resting CD4+ T cells [4,8,9]. In these early studies, the first phase lasted for about 6–11 days, and the second phase started after a viral load drop of 93%-99% from baseline, implying that the long-lived infected cells contributed ~1–7% of the total virus before treatment [4].

**Fig 1:**
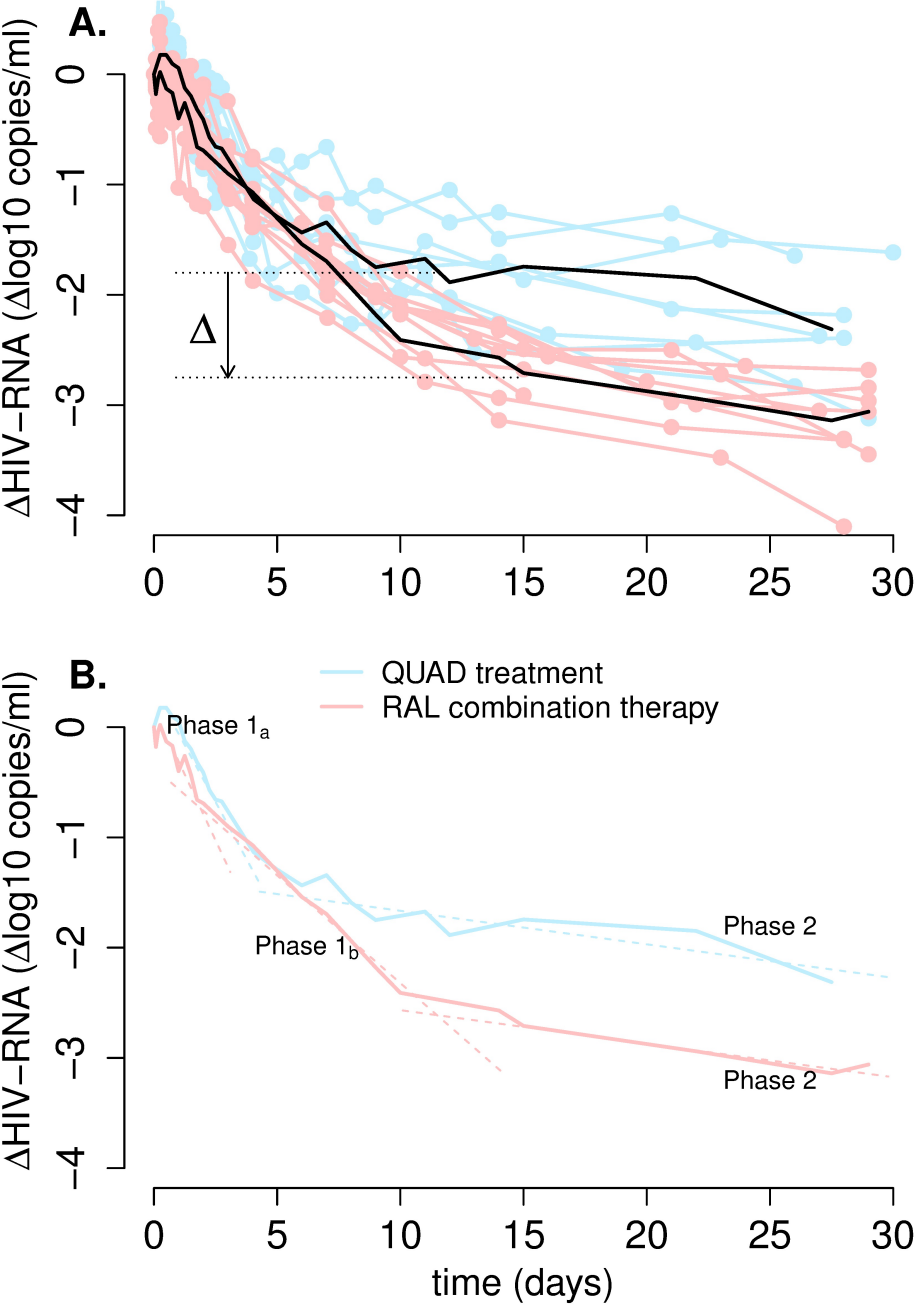
Viral dynamics after the initiation of treatment with or without RAL. (A) Blue and pink lines show the viral load relative to baseline for study participants in the QUAD treatment and RAL-combination therapy, respectively. Black lines represent the median values for each group. In the last phase, between about day 15 and day 30, the viral load in the RAL-combination participants is ~1-log lower (Δ~90% reduction) than the viral load in the quad-therapy group participants. (B) The median viral load profiles for each group present two phases of decay (1a and 2), but in addition the RAL-combination therapy includes an intermediate phase, 1b.

Different classes of drugs may lead to different patterns of viral decline, depending on where in the viral lifecycle the drug acts [10,11]. Modeling of one of the first clinical trials of HIV-1 treatment with an integrase strand transfer inhibitor (InSTI) showed that the viral load decline had first and second phase rates similar to those seen with InSTI-free regimens, with half-lives of ~1.2 days and ~15.5 days, respectively [12]. Although the data was sparse for the second phase, a unique characteristic of the viral load decline was that this phase started at much lower viral load levels than previously seen with InSTI-free regimens [12]. A recent study, with more frequent data, comparing InSTI-free and InSTI-containing regimens found that the rates of these two phases of decay were somewhat slower in the presence of an InSTI [13]. This study also confirmed that for InSTI-based regimens the viral load at the start of the second phase is ~1 log below the viral load at the start of the second phase for InSTI-free regimens [13] (Fig 1A). Another characteristic of viral decay under an InSTI is the separation of the first phase of decline into two sub-phases (phase 1a and phase 1b, Fig 1B) [10,14,15]. Analyzing the viral decay during the first 10 days of therapy with InSTI-containing regimens, we estimated [14] the half-life of the cells contributing to phase 1a as ~0.8 days, similar to the previously estimated half-life of short-lived infected cells (~0.7 days) [16]. In the same study, the half-life of the population of cells contributing to phase 1b was estimated as ~4.6 days, which is much shorter than the previously estimated half-life of the cells contributing to the second phase (~14 days) [4].

Several mechanistic models were proposed to explain the differences in the profile of viral decay between InSTI-free and InSTI-containing regimens [9,10,12,14,15,17], but a thorough comparison with appropriate data is still lacking. Here, we analyze in detail several data sets under InSTI-based and InSTI-free therapies using a unified approach. Our experimental data includes more frequent sampling throughout the first month of the viral load decline under InSTI-based therapy than any previous modeling study. We develop mathematical models that separate the pre-integration and post-integration state in both short-lived and long-lived infected cells. We then use a mixed-effect modeling approach to simultaneously fit the viral load data in InSTI-free combination drug therapy and therapies including raltegravir (RAL), an InSTI [13,14,16,18].

We discovered that the best explanation for all the available kinetic data is that short-lived infected cells have a fast integration rate and a short viral production period, while long-lived infected cells have slow integration, but an equally short viral production period. Thus, we attribute the long life span of some infected cells to slow proviral integration. We propose that both types of cells (short- and long-lived) represent mostly infected CD4+ T-cells but in activated and resting states, respectively.

## Results

### Viral load decay profiles

We analyzed a total of 47 participants in three HIV-1 treatment data sets (see Methods) [13,16,18]. Eight participants were treated with a 4-drug combination without InSTI (“quad-therapy) [16]; eleven participants were treated with a combination of RAL and two RTIs (“RAL-combination”); and twenty-eight people were treated with RAL monotherapy for 9 days (“RAL-monotherapy”).

Fig 1A presents the plasma viral load of all the participants from the quad-therapy and RAL-combination protocols, as well as the respective medians. This allows a clear visualization of the different patterns of decay under the two regimens (Fig 1B). In supplementary Figs B-D in S1 Text, we present the viral load data for all 47 individuals analyzed. Examination of these data shows that the second phase of viral load decline in individuals treated without RAL starts before than in participants under RAL-therapy (~ 5 days post-treatment initiation vs. ~10 days). At the start of the second phase, the viral load level in the RAL-combination participants is ~1-log lower than the viral load at the start of the second phase in the quad-therapy group (denoted by Δ in Fig 1A). We observe also that participants treated with RAL seem to have an intermediate phase of decline (phase 1b), which is slower than the first phase in the quad-therapy group, but faster than their second phase (Fig 1B). Finally, the slope of the last phase (phase 2) for RAL-combination therapy seems similar to the second phase slope for quad-therapy.

Thus, these data sets with more frequent measurements confirm the differences in viral load decay profiles between InSTI-containing and InSTI-free regimens presented before [12–14]. These observations of the differences between the two types of drug regimens, lead to four questions: i) Are these empirical observations borne out by rigorous statistical analyses? ii) Could the early second phase in the quad-therapy be due to the same mechanisms (*i*.*e*., decay of the same population of infected cells) as phase 1b in the InSTI-based treatment, which starts also at about ~5 days? iii) If not, what populations of infected cells contribute to each phase of decay in each type of treatment? And iv) Why do the second phases of decay seem have the same slope, but occur at very different viral load levels in the two types of regimens? We now answer these questions in turn.

### Statistical analyses of viral load phases of decay

To put the observations of the previous section in a rigorous footing, we used a mixed-effects approach [19] using two empirical models, allowing for two or three exponential decays, respectively (see Methods). Two exponentials correspond to the two phases of decay (as it seems to be the case in the quad-therapy) and three exponentials correspond to three phases of decay (phase 1a, 1b and 2). We fitted these two models to the viral load of the three data sets simultaneously to assess which model is more consistent with the data.

We found that the three-exponentials model did not fit the quad-therapy statistically better than the two-exponentials model (*p* = 0.89), while for the RAL-containing regimens the reverse is true (*p*<0.0001). Moreover, the initial decay rate was not significantly different between the treatment protocols, but the second decay rate was significantly different between the RAL-containing regimens (phase 1b) and the quad-therapy (phase 2) (*p* = 0.0007), with estimates of ~0.15 day^-1^ and ~0.05 day^-1^, respectively (Table 1). The estimate for the third decay rate in the RAL-combination arm (phase 2) was ~0.045 day^-1^ (Table 1), which is very similar to the phase 2 decay rate in the quad-therapy (~0.05 day^-1^).

**Table 1:** Comparison of the viral load decay rates during treatment with or without RAL. Rows represent each phase of decay and columns the estimates for each treatment using two methodologies: a heuristic multi-exponential model (two columns on the left) and the SRI model in Fig 2B and Eq (**1**) (two columns on the right). The estimation of the rate for phase 1b is only applicable in the case of treatment with RAL. All values are in units of day^-1^. For the SRI model, we also indicate the parameter combination defining the decay rate of each phase.

		Exponential models		Viral kinetic SRI model	
		RAL	No-RAL	RAL	No-RAL
**Phase 1**	**1a**	0.99	0.86	*δ*_*2*_ = 0.85	*δ*_*2*_ = 0.85
	**1b**	0.15		*δ*_1_+(1-*ω*)*k* = 0.39	
**Phase 2**		0.045	0.05	*δ*_*M*1_+(1-*ω*)*k*_1_ = 0.02	*δ*_*M*1_+*k*_1_ = 0.04

**Fig 2:**
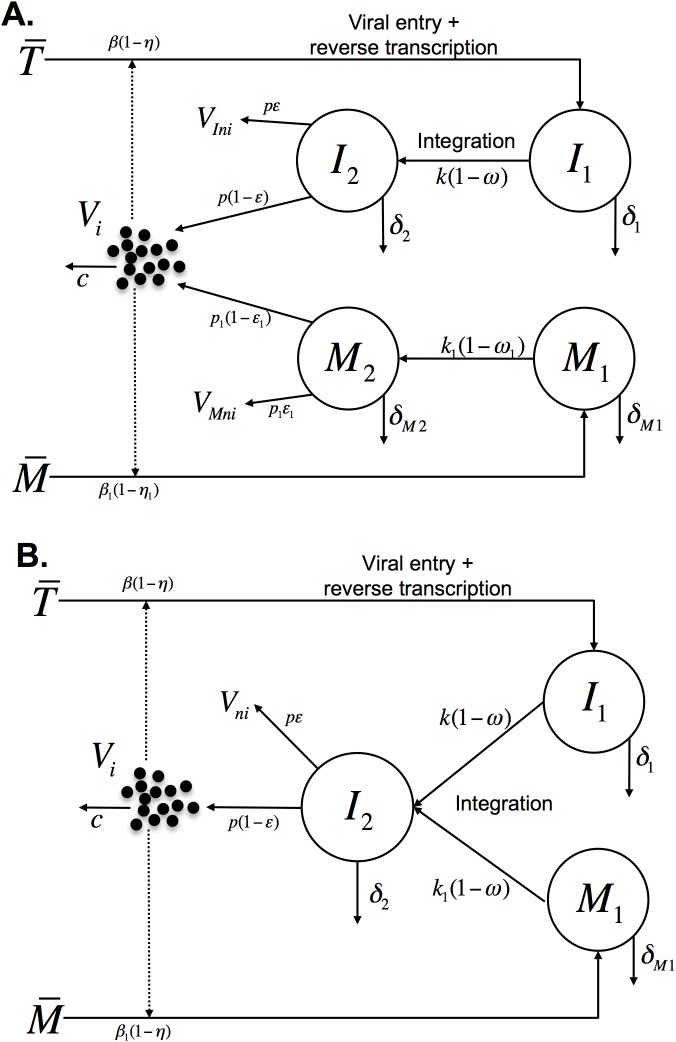
Schematics of the models. (A) The standard model with pre- and post-integration phases of infection. We follow two types of target cells that after infection will be short-lived, $\bar{T}$, or long-lived, $\bar{M}$. Target cells, $\bar{T}$, are infected by infectious virus, *V_i_*, at rate $\beta \bar{T}V_{i}$. The infection can be blocked by the activity of RTIs with effectiveness *η*. These infected cells, *I*_1_, are lost at rate *δ*_1_, or can undergo provirus integration at rate *k* and become productively infected cells *I*_2_. InSTIs block integration with efficacy *ω*. Cells with integrated provirus, *I*_2_, are lost at rate *δ*_2_. Virions are produced by these cells at rate *p* per cell and are cleared from the circulation at rate *c* per virion. Protease inhibitors block the production of infectious virus *V*_*Ii*_, and lead to production of non-infectious virus *V*_*Ini*_, with efficacy *ε*. The subscripts *I* and *M* are used to distinguish virions produced by short-lived and long-lived infected cells, respectively. The dynamics of long-lived cells are similar, but possibly with different rates as indicated. (B) The slow and rapid integration (SRI) model. The SRI model proposes that both short-lived cells with fast integration (*I*_1_) and long-lived cells with slow integration (*M*_1_) generate productively infected cells that die quickly (*I*_2_) (i.e. *δ*_2_ = *δ*_***M*2**_**)**.

These statistical results, thus, indicate that it is likely phase 1b for the RAL-containing treatments has a different viral source from phase two in the treatment without RAL. To explore the potential different populations of infected cells contributing to the viral load, we next analyzed the data in the context of mechanistic models of viral dynamics. We generalized a model developed to analyze the effects of RAL [14] by including the effect of reverse transcriptase and protease inhibitors and a compartment of long-lived infected cells, as illustrated in Fig 2A (details in Methods).

### Phase 1b is only observed in the presence of an InSTI

To understand whether phase 2 (without RAL) and phase 1b (with RAL) have the same origin, we used the mechanistic model described by Eq (1) in Methods, without long-lived cells [10,14] (see section 2 in S1 Text).

The model shows that there are two conditions that must be satisfied to observe two early phases of decay (*viz*. phase 1a and 1b): i) RAL must be present and ii) the efficacy of RAL must be above a critical threshold (*ω*>*ξ*(*δ*_1_+*k*)/(*δ*_2_+*k*), where *ω* is the efficacy of RAL, *ξ* is the combined efficacy of RTIs and PIs, *δ*_1_ and *δ*_2_ are the loss rates of short-lived infected cells before and after integration respectively, and *k* is the integration rate in these cells), but less than 100%. In the case of RAL monotherapy, *i*.*e*., *ξ = 0*, the value of the critical threshold is 0, and thus with monotherapy one should always see two early phases of decay (section 2 in S1 Text) [15]. In the case of therapy without an InSTI, *ω* = 0 and the model predicts (see section 2.2 in S1 Text) that after a short delay (so-called shoulder phase) only one exponential-like decay is observed early on, *i*.*e*., during phase one [9].

The interpretation of these results is that in the presence of RAL, the loss of cells in the pre-integration stage is uncovered (phase 1b), whereas without RAL, integration proceeds so fast that it is not possible to observe the loss of those cells (*i*.*e*., there is no phase 1b). Thus, to explain the second phase one needs another source of infected cells (*e*.*g*. long-lived HIV-infected cells).

### A subset of cells with slow integration is responsible for the phase 2 of viral load decline

In the RAL-containing regimens, phase 1 (phases 1a and 1b together) lasts longer (~10 days) than phase 1 during quad-therapy (~5 days). Consequently, for RAL-containing regimens phase 2 starts at a later time and at a lower viral load (Fig 1 and [12,13]). But why does this happen?

To investigate this issue, we used the model presented in Eq (1) (Fig 2A) and fitted it to the three datasets simultaneously. We found for the best fits that the rate of integration in the “long-lived cells” was very slow (*k*_1_<0.05 day^-1^) when compared to that of “short-lived cells” (*k* = 2.6 day^-1^). In addition, the estimate of the loss rate of long-lived cells producing virus (*δ*_*M*2_ ~0.90 day^-1^) was very close to the corresponding rate in short-lived cells (*δ*_2_ = 0.86 day^-1^). This suggests the hypothesis that “long-lived cells” have slow integration leading to their long lifespan, but after becoming productively infected they are lost at a rate equal to that of short-lived cells, *i*.*e*., *δ*_*M*2_ = *δ*_2_. Indeed making this assumption gave the best fitting model (with lowest AIC, see Table C in S1 Text). Thus, in the best model to describe the data, we have one population of productively infected cells with a short lifespan, which is generated from two populations, one with fast integration events and another with slow integration events (Fig 2B). In what follows we present analyses of the data using this model, which we call the SRI model (Slow and Rapid Integration model) to differentiate from the standard model in Fig 2A.

The SRI model has three compartments (Fig 2B), corresponding to cells with fast integration (*I*_1_) that quickly become productively infected (*I*_2_) and cells with slow integration (*M*_1_), which eventually also become productively infected (*I*_2_). In this new interpretation, the previous *M*_2_ compartment in the standard model is simply the subpopulation of productively infected cells (*I*_2_) that were generated from slow integration events (*i*.*e*., from *M*_1_), but it does not correspond to a physiologically separate population of cells.

**Fig 3:**
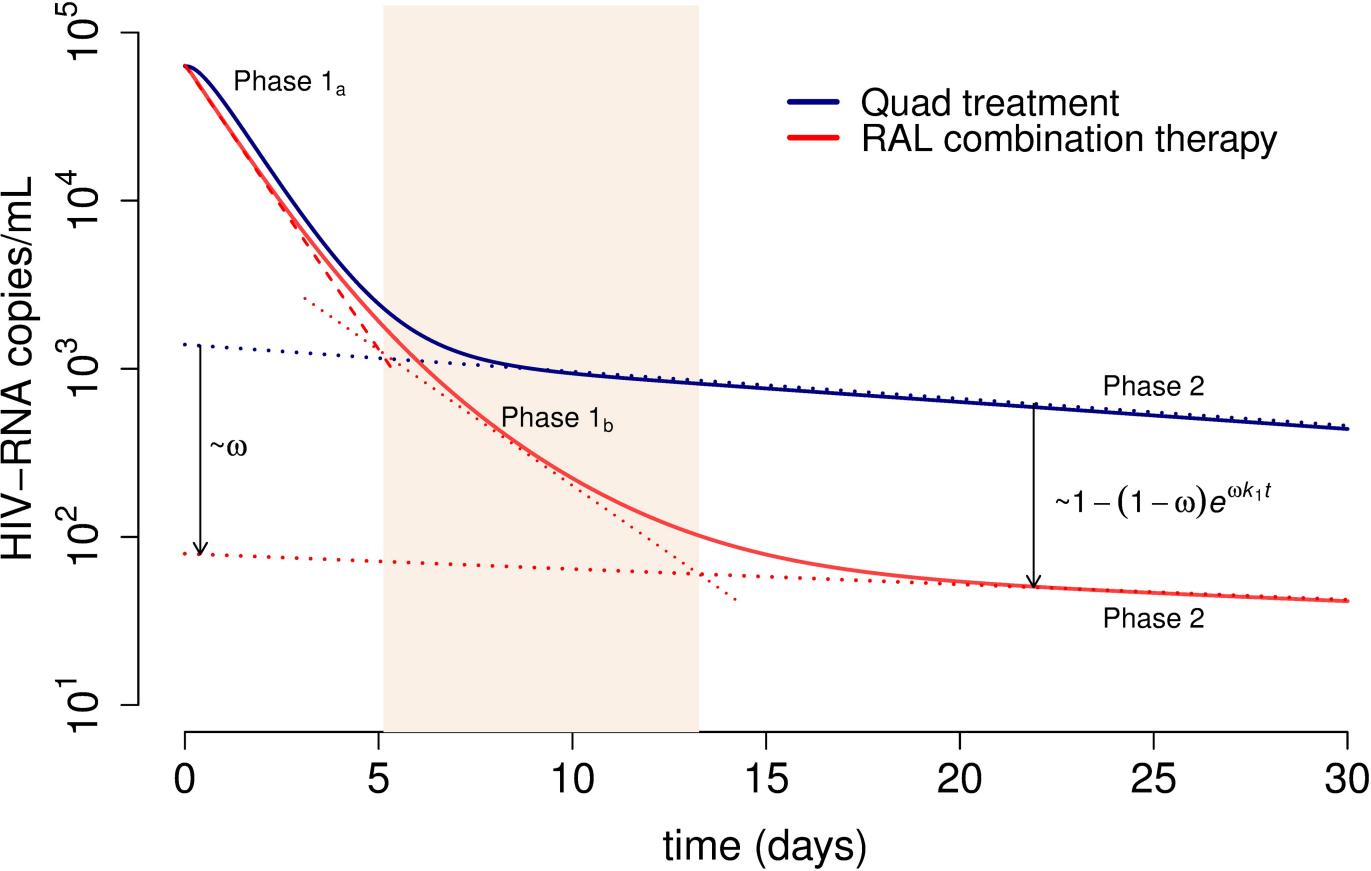
Predicted viral load decay for the quad and RAL-combination treatments using the best fit of the SRI model (Eq (1) when *δ*_2_ = *δ*_*M*2_) to the data. The estimated population parameters for each treatment group where used to plot the viral load decline under the effect of RAL+RTI (red) and quad therapy (blue). The dotted blue and red lines show the analytical approximation for the second phase of decay for quad therapy and RAL-combination therapy, respectively (see equation S.14 in S1 Text). The shadowed section highlights phase 1b for RAL-combination therapy. The viral load at the start of phase 2 in patients under RAL-based therapy is reduced with respect to the corresponding level in RAL-free therapy by a factor of $1-(1-\omega )e^{k_{1}\omega t}$. We fixed the values of the following parameters (see text for details): *V*_*I*_(0)/*V*(0) = 0.98, *δ*_*M*1_ = 0.02 day^-1^, *k* = 2.6 day^-1^ and *c* = 23 day^-1^ based on previous studies [14,16,39]. In addition, for RAL combination we used *η* = 0.95, *ε* = 0 and *ω* = 0.94, and for the quad therapy *η* = *ε* = 0.95 and *ω* = 0 [14]. The estimated best-fit population parameters are (estimated standard deviation in parenthesis): *δ*_1_ = 0.23 (0.04) day^-1^, *k*_1_ = 0.017 (0.01) day^-1^, *δ*_2_ = 0.85 (0.07) day^-1^ and *V*(0) = 4.8 (0.07).

**Fig 4:**
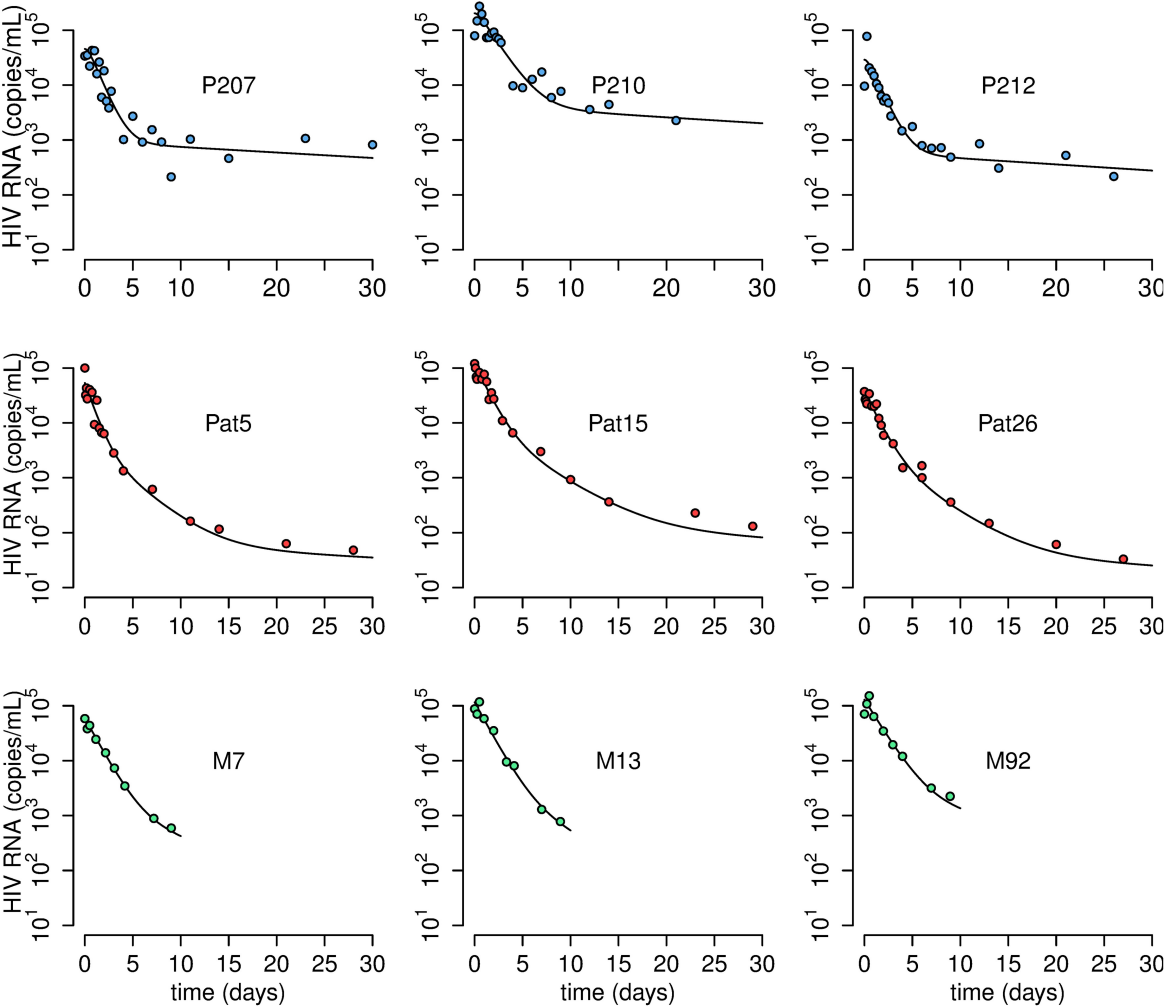
Representative individual fits for the three datasets. Blue, red and green circles represent HIV RNA measurements for the quad-based-, RAL-combination- and RAL-mono therapy, respectively. Solid black lines represent best fits from the SRI model using the mixed-effects approach. Parameter estimates for each individual are presented in Table E in S1 Text.

Fig 3 shows the predicted population viral load profiles for each treatment protocol resulting from fitting the SRI model to all the data simultaneously, and Table 1 summarizes the corresponding parameters. In Fig 4, we show best fits of the SRI model to the viral load data for representative individuals from the three treatments protocols. Individual best fits for all participants are shown in Figs B-D in S1 Text and the estimated parameters in Table E in S1 Text.

Overall, we found that the first phase of decline, which is phase 1 or 1a without or with RAL, respectively, corresponds to the loss of short-lived productively infected cells (*~δ*_2_, t_½_~ 20 h). Phase 1b only exists in RAL-based therapy and corresponds to the decay of short-lived cells pre-integration (at rate *~δ*_1_+(1-*ω*)*k*, t_½_~ 1.8 days). Phase 2 corresponds to the decay of long-lived cells pre-integration (at rate *~δ*_*M*1_+(1-*ω*)*k*_1_), which in the absence of RAL (*ω* = 0) is slightly faster (t_½_ ~ 19 days) than in the presence of RAL (t_½_ ~ 33 days), as RAL slows the loss of these cells by integration. Note that this estimated half-life in the absence of RAL is consistent with previous estimates (~14 days, range 6 to 24 days) [4,13]. Also, the difference in the second phase slope between the treatment regimens in the absence and presence of RAL (0.037 day^-1^ vs. 0.021 day^-1^) is sufficiently small that over 30 days of treatment it can’t be discerned in the figures (Fig 3).

From the best-fit parameters, we can calculate the relative proportion of infection events leading to infected cells with fast and slow integration (see section 3.1 in S1 Text). We estimate that ~96% of infections result in cells with fast integration (*I*_1_) and only 4% generate infected cells with slow integration (*M*_1_). Yet, due to the long-lived nature of the latter population, before treatment the pool of cells with slow integration (*M*_1_) represents about 40% of total infected cells in the SRI model (*i*.*e*., *M*_1_ is 40% of *I*_1_+*M*_1_+*I*_2_).

These results offer for the first time a consistent picture for the three treatment scenarios based on the same mechanistic model. However, we still need an explanation for the observed lower viral load during the second phase decline in InSTI-based therapies.

### The lower viral load of phase 2 under RAL-combination therapy depends on the efficacy of the integrase inhibitor

Based on the SRI model, we can directly calculate the reduction in viral load during the second phase with RAL treatment with respect to the viral load in the second phase with quad therapy. We find that this reduction is approximately $1-(1-\omega )e^{\omega k_{1}t}$ (section 3.2 in S1 Text). For example, at 12.5 days post-treatment, *i*.*e*. the beginning of phase two in the RAL-containing regimen, the viral load in the RAL-combination therapy is reduced by ~93% with respect to the viral load in the quad-therapy group (Fig 3). Interestingly, if we linearly extrapolate the second phase of decay for therapy without and with RAL back to time 0, then this reduction in viral load is given by ~*ω* (Fig 3). Therefore, our model provides a way to directly estimate the efficacy of RAL in blocking integration, *ω*, when compared to a highly-potent therapy without RAL, such as the quad-treatment. Thus, the SRI model intrinsically explains this difference in viral loads, based on the dynamics of different infected cell populations, and without the need for any extra assumptions.

Still, it would be important to have some intuitive understanding why there is a lower level of virus at the beginning of phase 2 in RAL-based regimens. In the SRI model, productively infected cells (*I*_2_) generate the observed virus. The different phases of virus decay correspond to loss of different cell populations contributing to generating those productively infected cells. In more detail and as shown in Movie S1, viral decay is observed as follows. Initially productively infected cells (*I*_2_) are lost quickly (rate *δ*_2_), corresponding to phase 1 or phase 1a, depending on the type of treatment. At the same time, cells progressing through integration (*I*_1_ and *M*_1_) replenish the productively infected cells (*I*_2_). In the presence of RTIs and PIs without InSTIs, the pool of cells with fast integration (*I*_1_) is quickly exhausted. In this case, the second phase can be observed when the main contribution to productively infected cells (*I*_2_) comes from the conversion of slowly integrating cells (*M*_1_) at rate *k*_1_ into productively infected cells. The productively infected cells then have an effective decay rate given by (~*δ*_M1_+*k*_1_), which is the rate limiting step. Paradoxically, in the presence of an InSTI, the pool of cells that can integrate fast (*I*_1_) is exhausted more slowly, because integration is blocked/slowed but not prevented completely. This results in phase 1b with slope (~*δ*_1_+*k*(1-*ω*)) due to the slow decay of cells in *I*_1_. Together phases 1a and 1b last longer than phase 1, because it takes longer to lose most cells in *I*_1_. Again, as in the quad-therapy, the second phase is observed when the main contribution to productively infected cells comes from the slow integrating cells (*M*_1_), which in the presence of an InSTI occurs later, because integration is slowed in this compartment too. This explains the lower viral load level at the start of the second phase with an InSTI regimen. All the infected cell populations are decaying from early on at the start of treatment, but the observed viral load reflects the decline of different populations in turn, as the populations with faster turnover are lost. However, it raises another question, why does the second phase in the quad-treatment start so early (~5 days), even earlier than what had been observed before in RTI+PI treatments?

### The time of the start of the second phase without an InSTI depends on the efficacy of the drug regimen

In the quad-therapy data set the first phase lasted only ~5 days, whereas early studies with less potent RTIs and PIs indicated that this phase lasted for about 6 to 11 days [4]. Using the SRI model, we found that the higher the effectiveness of the RTI+PI regimen (*i*.*e*., the larger the effectiveness of the RTI, *η* and of the PI, *ε*), the earlier the switch from the first to the second phase (Fig E(a) in S1 Text). In fact, we can calculate (Eq. S15) that the difference in the times of transition from ~8 days in the earlier studies to 5 days with the quad-regime implies that the latter had an efficacy ~1.6-fold higher, *i*.*e*., early therapies had an efficacy *ξ* ~ 0.62. This result compares favorably with a previous study that indicated that early therapies had an efficacy ~75% of the quad-treatment protocol [20]. In the quad regimen with higher efficacy, the transition from the first to second phase occurs earlier and at a slightly lower viral load, because of faster viral decay (Fig E(b) in S1 Text), as discussed in [16]. Thus, the SRI model clearly explains the early transition to the second phase in the quad-therapy regimen is due to the high efficacy of the combination treatment.

## Discussion

Analysis of viral dynamics under different treatment regimens provides insight into the possible virus sources for the observed phases of plasma viral load decay. Under RAL-containing regimens the original phase 1 decay described for PI and RTI containing regimes [4,5] is replaced by two phases, called 1a and 1b [10,14,15], which are then followed by a second phase that starts at lower viral load levels than the start of the second phase with InSTI-free regimens due to the presence of phase 1b. Here we presented for the first time a detailed quantification of the dynamics of cell subpopulations contributing to the observed viral kinetics over the first month of treatment. There are other slower phases of decay at later times, which are not studied here [3,21,22].

The mechanistic reasons for the peculiar viral load decay profile under InSTI regimens have been the subject of previous studies. Murray *et al*. [12] first developed models to analyze the viral load decay kinetics under RAL. The models presented in [12] are different from our SRI model, they were not fit to individual patient data, and they cannot recapitulate the phase 1b observed in the data. However, the models in [12] that could qualitatively explain the data had the interesting characteristic that the second phase virus was produced from the same cell population as the first phase virus. The difference was in the infection kinetics (e.g., proviral integration), which could be fast (in short-lived cells) or slow (in long-lived cells). This idea echoes our final model where we found that the death rate of short-lived cells was the same as the death rate of long-lived cells after they both start producing virus (*i*.*e*., *δ*_M2_ = *δ*_2_).

Sedaghat *et al*. [9,17,23], Gilmore *et al*. [10] and Wang *et al*. [15] presented detailed mathematical analyses of models similar to ours. However, they did not analyze clinical data and/or had only very sparse viral load data, and, thus, had to make various assumptions about parameters and their relationships. For example, Sedaghat *et al*. [9,17,23] studied without fitting data the features of the second phase of viral decline. They concluded that in the most likely scenario the loss rate of long-lived infected cells pre-integration (~*δ*_*M*1_+*k*_1_) should be greater than the loss rate of long-lived productively infected cells (~*δ*_*M*2_); and that the latter should be the slope of the second phase (equal) in both treatment cases (with and without integrase inhibitors). By quantitative analyses of viral load data and fitting, we rather found that *δ*_*M*2_>*δ*_*M*1_+*k*_1_ even when using the conclusions in Sedaghat *et al*. [9,17,23] as initial guesses in the fitting procedure. Thus, we now show that the second phase slope is the loss rate of long-lived cells pre-integration, *i*.*e*., *~δ*_*M*1_+(1-*ω*)*k*_1_, and that this slope is slightly different with and without an integrase inhibitor, as also found previously by simple linear regression [13]. Interestingly, Sedaghat et al. briefly considered an “intriguing possibility” consistent with our results that the loss rate of long-lived productively infected cells is the same as that for short-lived productively infected cells, but dismissed it [17].

Our detailed data-driven comparison between treatment regimens with and without RAL leads to a novel interpretation of the profile of HIV-1 decay under treatment. The existence of an early (fast) and a late (slow) phase of viral decay is explained, as before, by decay of two “different” cell populations. However, the best fit of the SRI model supports the idea that the dynamics of proviral integration distinguishes these two cell populations, but they both could be CD4+ T-cells. The early fast decay in viral load (including phase 1a and 1b) is most likely due to infection of activated CD4+ T-cells, which quickly progress to viral production. The slow late decay (phase 2) is likely due to infection of resting CD4+ T-cells, which then progress slowly to provirus integration. This interpretation is consistent with data indicating that in resting CD4+ T-cells integration proceeds slowly [24–26]. In the SRI model a fraction of these cells eventually complete integration and become productively infected cells. The resting cells have a slow loss rate before integration, thus they are long-lived, but we found that after becoming productively infected they have a high loss rate, similar to short-lived productively infected cells. One explanation for this result could be that some stimulus activates these cells, which then quickly integrate and start producing virus. Although this interpretation of the data may be surprising, with hindsight, one recognizes that the differences observed in viral load decay profiles between treatments with and without RAL must be due to some process involving proviral integration, which is the step of the lifecycle blocked by the drug. We note that this interpretation is unlikely to depend on the assumptions of the SRI model, which we tested by examining other models and parameter ranges without finding any important effect (section 4.4 in S1 Text). In particular, alternative models, where the second phase is due to cells with virus already integrated that are lost slowly, does not generate viral decay profiles consistent with those observed. Indeed, we started our analysis with this assumption (Fig 2A) and found that the resulting differences in viral loads in the second phase between treatments with or without RAL were too small compared with the data (see section 4.3 and Fig A in S1 Text).

We estimate that before treatment, the vast majority of virus is produced by short-lived cells [4], which is indicated by the large decay in viral load during the first phase, and only a small fraction of virus is produced from cells with slow proviral integration. Several recent reports have indicated that non-integrated HIV DNA in resting CD4+ T-cell is capable of integration and generating productive infection upon activation [24,27–32]. Indeed, this phenomenon has been dubbed “pre-integration latency” [7]. Here we also find that the loss of cells in this pre-integration stage in the absence of InSTI therapy is very slow (*t*_1/2_ ~ 19 days), consistent with a study measuring the decay of unintegrated HIV DNA in chronically infected patients under successful HAART that reported a half-life of 26 days [33]. Some studies have described a very rapid decay of the pre-integration complex (PIC) with half-life of ~1 day in resting cells *in vitro* [24,28]. However, there are other studies that report stable accumulation of HIV DNA transcripts over longer timespans [34] with inducible pre-integration latency over times up to 28 days *in vitro* [27,30]. It has also been suggested that *in vivo* the half-life of pre-integration latent cells can be longer than *in vitro* [35]. Moreover, recent evidence has shown that 2-LTR circles, which are very stable, can be cleaved by the viral integrase forming a viable substrate for integration and rescuing viral production [29]. Altogether, it is possible that *in vivo* resting infected cells in the pre-integration stage have a broad distribution of half-lives. Thus, although the half-life of “pre-integration latency” is somewhat controversial, we think that our results add a new piece of evidence to indicate that *in vivo* on average this half-life is long and physiologically relevant, since these cells can be activated into producing virus.

An alternative explanation invoked for the second phase of viral decay is that the long-lived cells are infected macrophages [4,9,23]. Some results indicate that HIV DNA integration is slow in primary blood monocytes [36], however other studies have measured fast integration (~3.4 hours) [23]. In any case, it is thought that infected macrophages are long-lived even after starting to produce virus [7], which is inconsistent with our findings. Moreover, it is unlikely that infected monocytes with unintegrated HIV DNA form as large a pool of infected cells as estimated here [37]. However, it is possible that in addition to the cell compartments in the SRI model, a compartment of long-lived productively infected cells, such as macrophages (Fig 2A), exists. The contribution of these cells to the second phase in viral load should be minimal and, thus, it would not affect the results presented by the SRI model.

Altogether, the most parsimonious interpretation of the analyses of these detailed data sets is that the slow second phase viral decay corresponds to the loss of infected cells with unintegrated provirus (possibly resting CD4+ T-cells) by death, by loss of the pre-integration intermediates, or by integration of the HIV DNA to generate productively infected cells. This description is valid both in regimens with and without an InSTI, and thus alters the interpretation of the second phase of viral decay presented in previous studies [4,9,17]. Furthermore, our results explain why the second phase decay is slightly slower in the RAL containing regimen than in the RAL-free regime, as found previously [13]. This difference is small because it depends on the integration rate *k*_1_, which is slow.

In conclusion, we have proposed a new model that explains the main differences of viral load decline under InSTI therapy (as prototyped here by RAL) when compared with InSTI-free regimens: i) the two early phases 1a and 1b of viral decay; ii) the ~1 log lower viral load at the start of the second phase; and iii) the nearly identical second phase slope with and without an InSTI. Fitting this model to frequently sampled viral load data indicates that the second phase of viral decay is due to a subset of cells completing integration very slowly, which most likely are infected resting CD4+ T-cells that eventually complete integration to produce virus and then die quickly.

## Methods

### HIV-1 treatment clinical data

The first set of data is from a trial with a highly active antiretroviral quad-regimen [16], where nine chronically HIV-1-infected individuals were treated with the boosted PI lopinavir-ritonavir (1,066 and 266 mg/day, respectively), and the RTIs efavirenz (600 mg/day), lamivudine (300 mg/day), and tenofovir DF (300 mg/day). Plasma viral loads were measured by RT-PCR (Roche Amplicor Ul- trasensitive Cobas 1.5) with a limit of quantification (LoQ) of 50 copies/ml at 6 h intervals during the first 72 h, then daily until day 10, and then weekly until day 28 [16]. One participant could not be analyzed, because the precise time of each viral load quantification was not available. We refer to this data set as the “quad treatment” data.

The second data set is from 28 HIV-1-infected participants treated with RAL monotherapy for 9 days [18]. Participants received 100, 200, 400, or 600 mg RAL twice daily, and had plasma HIV RNA measured before the first dose, 6 and 12 hours post-dose, and on days 1, 2, 3, 4, 7, 9. Plasma was assayed for HIV-1 RNA using the Amplicor HIV-1 Monitor Assay, version 1.5 with a LoQ = 400 copies/ml. If the result was below that level, the UltraSensitive HIV-1 Monitor Assay (LoQ = 50 copies/ml) was used (Roche Molecular Diagnostics, Alameda, CA). We treated plasma HIV RNA data below the limits of quantification as censored at the corresponding values. Because no difference in the pattern of decay or efficiency between the different doses was found [14,18], we analyze all doses together. We refer to this data set as the “RAL-monotherapy” data.

The third set of data was obtained from 11 HIV-1-infected participants treated with a combination of FTC/TDF 200 mg/300 mg daily plus RAL 400 mg twice daily enrolled in the intensive viral dynamics A5249s substudy of ACTG protocol A5248 [13]. Plasma HIV-1 RNA was measured at baseline, and at 2, 4, 6, 12, 18, 24, 30, 36, 42, and 48 hours and days 3, 4, 7, 10, 14, 21 and 28 after treatment initiation. Plasma was assayed for HIV-1 RNA using the Amplicor HIV-1 Monitor, version 1.5, UltraSensitive protocol (LoQ = 50 copies/mL; Roche Molecular Systems, Branchburg, NJ). We refer to this data set as the “RAL-combination therapy” data.

The study protocols were approved by the respective institutional review board at each of the participating clinical research sites, and all subjects provided signed informed consent (see details in [13,16,18]). All data analyzed were anonymized.

### Mathematical model of viral infection and treatment

We first analyzed the data with double and triple exponential decay curves, corresponding to sums of (*j* = 2 or *j* = 3) terms of the form $C_{j}e^{-\alpha_{j}t}$, to see if the data was consistent with two or three phases of decay and what the respective slopes (*α*_*j*_) were.

We then generalized our model developed to analyze the effects of RAL [14] by including the effect of protease inhibitors and a compartment of long-lived infected cells, to account for the long-term (up to 30 days) follow-up. The model is a modification of the standard model of virus dynamics [1,3] separating the compartment of infected cells in pre-integration and post-integration states in both short-lived and long-lived infected cells. A schematic of the model is shown in Fig 2A, and the full system of differential equations describing the dynamics is given in the S1 Text (section 1). We assumed that each drug has the same efficacy in short and long-lived infected cells and that the dynamics of virus is much faster than that of infected cells. Both of these assumptions have been widely used before [4,5,9,14,15,17], and result in the simplified model

$$\begin{array}{l} \frac{d{\hat{I}}_{1}}{dt}=(1-\xi )\hat{T}V-\delta_{1}{\hat{I}}_{1}-k(1-\omega ){\hat{I}}_{1}\\ \frac{d{\hat{M}}_{1}}{dt}=(1-\xi )\hat{M}V-\delta_{M1}{\hat{M}}_{1}-k_{1}(1-\omega ){\hat{M}}_{1}\\ \frac{dV_{I}}{dt}=\frac{k}{c}(1-\omega ){\hat{I}}_{1}-\delta_{2}V_{I}\\ \frac{dV_{M}}{dt}=\frac{k_{1}}{c}(1-\omega ){\hat{M}}_{1}-\delta_{M2}V_{M} \end{array}$$ (1)

where *V*_*I*_ and *V*_*M*_ denote virus produced by short- and long-lived infected cells, *I* and *M*, respectively, total virus is *V* = *V*_*I*_ + *V*_*M*_, *ω* is the effectiveness of the InSTI and *ξ* is the combined effectiveness of the PIs (*ε*) and RTIs (*ε*) given by (1-*ξ*) = (1-*η*)(1-*η*). See section 1 in S1 Text for further details about the definitions of the variables and parameters. In particular, note that without loss of generality, we rescaled the variables so that the rate of infection, which in our model includes reverse transcription, and the viral production rate no longer appear in the equations, although they can be different between short-lived, *I*, and long-lived, *M*, cells.

### Data fitting

We fitted the model to the plasma HIV-1 RNA data using non-linear mixed effects (NLME) models. In this approach, we represent the parameters for each individual (*i*) as *μ*_*i*_ = *θe*^*φ_i_*^, where *θ* is the median value of the parameter in the population, and *φ*_*i*_ the random terms, which are normally distributed with zero mean and a variance to be estimated. We fitted the model in Eq (1) simultaneously to the three sets of data using the software MONOLIX (www.lixoft.eu), to estimate the population parameters (by maximum likelihood) and the variances of the random effects. In total, we fit 613 data points of 47 participants simultaneously to estimate between 4 and 6 parameters, and their corresponding variances in various versions of the model. For each model fit, we estimate the log-likelihood (log *L*) and compute the Akaike Information Criteria (AIC = -2log *L*+2*m*, where *m* is the number of parameters estimated) [38]. We compared the AIC for different model assumptions and computed ΔAIC, the difference in AIC between the best model and other model scenarios analyzed. We assumed models had similar support if their ΔAIC<2 [38]. P-values were based on the log-likelihood ratio test and significance was assessed at the *α* = 0.05 level.

To implement the fitting, we first explored the possible range of values for the new parameters in our model, *δ*_*M*1_, *δ*_*M*2_ and *k*_1_ describing long-lived infected cells (see Fig 2A), keeping fixed the remaining parameters based on estimates from previous studies [14,16,39], and obtaining the values of $\hat{T}$ and $\hat{M}$ from steady state assumptions (see Eq. S.4). We performed Latin-hypercube sampling resulting in 64,000 (40 values for each parameter) simulations of the model in Eq (1) for different values of the parameters [40]. We looked for combinations of parameters that showed viral decay properties consistent with the observations in the data (section 4.1 in S1 Text). Namely, a reduction in the viral load greater than 70% at the start of the second phase of decline under RAL-combination therapy relative to start of the second phase in the quad-based treatment; and similar second phase slopes. We found that these biological conditions are only satisfied for small values of *δ*_*M*1_ (<0.07 day^-1^) and *k*_1_ (<0.08 day^-1^), along with values of *δ*_*M*2_ greater than 0.15 day^-1^ (Fig A in S1 Text). These results provided starting guesses for these parameters in the viral load fits. To simplify the fitting procedure and obtain convergence, we fixed five parameters (*ξ*, *k*, *ω*, *c*, and the fraction of virus produced from long-lived infected cells, see section 4.2 in S1 Text) at values previously estimated by us and others [14,39]. In addition, the values of $\hat{T}$ and $\hat{M}$ in Eq (1) were obtained from the other parameters based on pre-therapy steady state assumptions (see Eq. S.4 in S1 Text). Still, it was difficult to estimate independently both *δ*_*M*1_ and *k*_1_, since when one increased the other tended to decrease (see Tables A and B in S1 Text). Therefore, we performed the fitting procedure for different fixed values of *δ*_*M*1_, and found the best fits when *δ*_*M*1_ = 0.01–0.02 day^-1^, based on AIC (see Table B in S1 Text). We then estimated the remaining parameters: *V*_0_, *δ*_1_, *δ*_2_, *δ*_*M*2_ and *k*_1_.

## Supporting information

S1 Text Supporting information. Detailed analyses of the models presented in the text and further details of methodology and alternative models.
(PDF)

S1 Movie Animation of the dynamics predicted by the model S.13 for the best fits to the RAL+RTI (red) and QUAD therapy (blue) data. The panels at the top show in block diagrams the decay of the pre-integration cell compartments *I*_1_ and *M*_1_, and the productively infected cells compartment *I*_2_ in both, RAL combination therapy (red) and QUAD therapy (blue). Horizontal arrows indicate the start of the second phase for each therapy in each compartment. The bottom panel shows the corresponding decay in (log_10_) viral load over time, where the vertical dashed lines indicate the start of the second phase (QUAD therapy in blue and RAL-combination therapy in red). Virus is produced by productively infected cells (*I*_2_). Initially they are lost quickly by death (rate *δ*_2_), for both types of treatment. At the same time these cells (*I*_2_) are replenished by infected cells progressing through integration (*I*_1_ and *M*_1_). In the presence of RTIs and PIs without InSTIs, the pool of cells with fast integration (*I*_1_) decays quickly and the second phase starts when the main contribution to *I*_2_ comes from the conversion of slowly integrating cells (*M*_1_) at rate *k*_1_. The productively infected cells in the QUAD panel then have an effective decay rate given by (~*δ*_M1_+*k*_1_), which is the rate limiting step. In the presence of an InSTI, the pool of cells that can integrate fast (*I*_1_) decreases more slowly, because integration is slowed down but not prevented completely. This results in phase 1b with slope (~*δ*_1_+*k*(1-*ω*)) equal to the slower decay of cells in *I*_1_. Together phases 1a and 1b last longer than phase 1, because it takes longer to lose cells in *I*_1_. The second phase for RAL combination therapy starts when the main contribution to productively infected cells comes from *M*_1_, which in the presence of an InSTI occurs later, because integration is slowed down in this compartment too. This explains the lower viral load level at the start of the second phase with an InSTI regimen. Fixed parameter values are: *V*_*I*_(0)/*V*(0) = 0.98, *δ*_*M*1_ = 0.02 day^-1^, *k* = 2.6 day^-1^ and c = 23 day^-1^ based on previous studies [14,16,39]. In addition, for RAL combination we used *η* = 0.95, *ε* = 0 and *ω* = 0.94, and for the quad therapy *η* = *ε* = 0.95 and *ω* = 0. The estimated best-fit population parameters are (estimated standard deviation in parenthesis): *δ*_1_ = 0.23 (0.04) day^- 1^, *k*_1_ = 0.017 (0.01) day^-1^, *δ*_M2_ = *δ*_2_ = 0.85 (0.07) day^-1^ and *V*(0) = 4.8 (0.07).
(MP4)

S1 Dataset Quad-therapy data. Full quad therapy data used in the analyses described in the manuscript.
(CSV)

S2 Dataset RAL-combination therapy data. Full RAL-combination therapy data used in the analyses described in the manuscript.
(CSV)
