# Supplementary material for: Treatment with integrase inhibitor suggests a new interpretation of HIV RNA decay curves that reveals a subset of cells with slow integration
Source: PLoS Pathog. 2017 Jul 5;13(7):e1006478. doi: 10.1371/journal.ppat.1006478 (PMC5513547; doi:10.1371/journal.ppat.1006478)
Supplement: S1 Text — Detailed analyses of the models presented in the text and further details of methodology and alternative models. (PDF) [file ppat.1006478.s001.pdf]

Integrase inhibitor therapy suggests a new interpretation of HIV RNA decay curves that reveals a subset of cells with slow integration

S1 Text

Erwing Fabian Cardozo[1], Adriana Andrade[2], John W. Mellors[3], Daniel R. Kuritzkes[4], Alan S. Perelson[1], Ruy M. Ribeiro[1,5]

[1]Theoretical Biology and Biophysics, Los Alamos National Laboratory, Los Alamos, NM, USA
[2]The Johns Hopkins University, Baltimore, MD, USA
[3]University of Pittsburgh School of Medicine, Pittsburgh, PA, USA
[4]Brigham and Women's Hospital, Harvard Medical School, Boston, MA, USA
[5]Laboratório de Biomatemática, Faculdade de Medicina, Universidade de Lisboa, Portugal

1 Full Mathematical Model

We generalize the model we presented in Andrade *et al.*[1], by including the effect of protease inhibitors (generation of non-infectious viruses) and a compartment of long-lived infected cells. The purpose of the model is to study the characteristics of the second phase of viral load decay in the presence or absence of InSTI. In the model, we assume that target cells remain at an approximately constant level, $\bar{T}$, during the first month of infection, an approximation that is commonly used[1–3]. Target cells are infected by infectious virus, V_i, at rate $\beta\bar{T}V_i$. The infection can be blocked by the activity of RTIs with effectiveness η, where η=1 corresponds to complete blocking of infection. These infected cells, I_1, can be lost at rate δ_1 (by degradation of HIV DNA intermediates or death) or can progress with provirus integration at rate k. We include the effect of an InSTI in blocking integration with efficacy ω. Cells with integrated provirus are assumed to be productively infected cells, I_2, that die at rate δ_2. Virions are produced by these cells at rate p per cell and are cleared from the circulation at rate c per virion. Protease inhibitors block the production of infectious virus V_{Ii}, and lead to production of non-infectious virus V_{Ini}, with efficacy ε. Thus, we consider two classes of infected cells: I_1, cells in which reverse transcription has occurred, and I_2, cells after provirus integration, which are productively infected.

The dynamics of long-lived cells are similar. Uninfected cells, $\bar{M}$, are infected at rate $\beta_1\bar{M}V_i$, then enter a stage with unintegrated provirus M_1. These cells are lost at a rate δ_{M1}

and become productively infected cells, M_2, at a rate k_1. Finally, productively infected long-lived cells die at a rate δ_{M2}. The effects of RTIs and InSTIs are modelled in a similar way to those in short-lived infected cells, with efficacy η_1 and ω_1, respectively. Protease inhibitors block production of infectious virus V_{Mi}, and lead to production of non-infectious virus V_{Mni}, with efficacy ε_1. Thus, the total infectious virus V_i is given by $V_{Ii} + V_{Mi}$. Note that for clarity we separate the virus contributions from short- and long-lived cells, $V_I = V_{Ii}+V_{Ini}$ and $V_M = V_{Mi}+V_{Mni}$, respectively, although our results do not depend on this. In principle virus released from different cell types may be distinguishable based on the cell surface proteins the virions carry. The following system of differential equations describes these dynamics

$$
\begin{aligned}
\frac{dI_1}{dt} &= (1-\eta)\beta\bar{T}V_i - \delta_1 I_1 - k(1-\omega)I_1 \\
\frac{dI_2}{dt} &= k(1-\omega)I_1 - \delta_2 I_2 \\
\frac{dM_1}{dt} &= (1-\eta_1)\beta_1\bar{M}V_i - \delta_{M1}M_1 - k_1(1-\omega_1)M_1 \\
\frac{dM_2}{dt} &= k_1(1-\omega_1)M_1 - \delta_{M2}M_2 \\
\frac{dV_{Ii}}{dt} &= p(1-\varepsilon)I_2 - cV_{Ii} \\
\frac{dV_{Ini}}{dt} &= \varepsilon p I_2 - cV_{Ini} \\
\frac{dV_{Mi}}{dt} &= (1-\varepsilon_1)p_1 M_2 - cV_{Mi} \\
\frac{dV_{Mni}}{dt} &= \varepsilon_1 p_1 M_2 - cV_{Mni}
\end{aligned}
\qquad \textbf{(S.1)}
$$

Redefining the variables so that $\hat{I}_1 = pI_1$, $\hat{I}_2 = pI_2$, $\hat{M}_1 = p_1 M_1$, $\hat{M}_2 = p_1 M_2$, $\hat{T} = p\beta\bar{T}$ and $\hat{M} = p_1\beta_1\bar{M}$, we can re-write, without loss of generality, the previous system as

$$
\begin{aligned}
\frac{d\hat{I}_1}{dt} &= (1-\eta)\hat{T}V_i - \delta_1 \hat{I}_1 - k(1-\omega)\hat{I}_1 \\
\frac{d\hat{I}_2}{dt} &= k(1-\omega)\hat{I}_1 - \delta_2 \hat{I}_2 \\
\frac{d\hat{M}_1}{dt} &= (1-\eta_1)\hat{M}V_i - \delta_{M1}\hat{M}_1 - k_1(1-\omega_1)\hat{M}_1 \\
\frac{d\hat{M}_2}{dt} &= k_1(1-\omega_1)\hat{M}_1 - \delta_{M2}\hat{M}_2 \\
\frac{dV_{Ii}}{dt} &= (1-\varepsilon)\hat{I}_2 - cV_{Ii} \\
\frac{dV_{Ini}}{dt} &= \varepsilon \hat{I}_2 - cV_{Ini} \\
\frac{dV_{Mi}}{dt} &= (1-\varepsilon_1)\hat{M}_2 - cV_{Mi} \\
\frac{dV_{Mni}}{dt} &= \varepsilon_1 \hat{M}_2 - cV_{Mni}
\end{aligned}
\qquad \textbf{(S.2)}
$$

Note that this redefinition of variables does not change the predicted dynamics of the system, but it allows the reduction of the number of parameters (in particular p and β), which are absorbed into the steady state conditions (see below). It is expected that these rates are different in short- and long-lived cells, but they do not contribute to the observed behavior of the viral load.

Further, making the common assumption of quasi-steady state of the free virus, *i.e.*, that the dynamics of free virus is much faster than that of $\hat{I}_2$ and $\hat{M}_2$, we have $\hat{I}_2 \approx c(V_{Ii}+V_{Ini}) = cV_I$ and $\hat{M}_2 \approx c(V_{Mi}+V_{Mni}) = cV_M$, respectively. Moreover, with the same assumption and by adding $V_{Ii}+V_{Mi}$ and $V_{Ini}+V_{Mni}$, we also have that $\hat{I}_2(1-\varepsilon)+\hat{M}_2(1-\varepsilon_1) \approx c(V_{Ii}+V_{Mi}) = cV_i$ and $\hat{I}_2\varepsilon+\hat{M}_2\varepsilon_1 \approx c(V_{Ini}+V_{Mni}) = cV_{ni}$, respectively. Finally, we assume that $\omega=\omega_1$, $\varepsilon=\varepsilon_1$ and $\eta=\eta_1$, which has been used in multiple previous studies [1–6] (but see below for other assumptions). Putting all this together, one obtains $V_i \approx \frac{(\hat{I}_2+\hat{M}_2)}{c}(1-\varepsilon) = (V_I+V_M)(1-\varepsilon)$. Thus, by defining $V=V_I+V_M$, we get the simplified system

$$
\begin{aligned}
\frac{d\hat{I}_1}{dt} &= (1-\eta)(1-\varepsilon)\hat{T}V - \delta_1\hat{I}_1 - k(1-\omega)\hat{I}_1 \\
\frac{d\hat{M}_1}{dt} &= (1-\eta)(1-\varepsilon)\hat{M}V - \delta_{M1}\hat{M}_1 - k_1(1-\omega)\hat{M}_1 \\
\frac{dV_I}{dt} &= \frac{k}{c}(1-\omega)\hat{I}_1 - \delta_2 V_I \\
\frac{dV_M}{dt} &= \frac{k_1}{c}(1-\omega)\hat{M}_1 - \delta_{M2} V_M
\end{aligned}
\qquad \textbf{(S.3)}
$$

The steady state of Eq. (S.3) before therapy, *i.e.*, when $\varepsilon=\eta=\omega=0$, with $\bar{V}=\bar{V}_I+\bar{V}_M$, the pre-therapy viral load steady state, is

$$
\begin{aligned}
\bar{\hat{T}} &= \frac{c\delta_2(k+\delta_1)\bar{V}_I}{k\bar{V}} \quad \& \quad \bar{\hat{M}} = \frac{c\delta_{M2}(k_1+\delta_{M1})\bar{V}_M}{k_1\bar{V}} \\
\bar{\hat{I}}_1 &= \frac{c\delta_2}{k}\bar{V}_I \quad \& \quad \bar{\hat{M}}_1 = \frac{c\delta_{M2}}{k_1}\bar{V}_M
\end{aligned}
\qquad \textbf{(S.4)}
$$

2 Mathematical model without long-lived cells

To study short term dynamics of viruses, say within the first 10-12 days, we can disregard long-lived cells [1]. In this case, $V=V_I$. The model in Eq. (S.3) simplifies to,

$$
\begin{aligned}
\frac{d\hat{I}_1}{dt} &= (1-\eta)(1-\varepsilon)\hat{T}V - \delta_1\hat{I}_1 - k(1-\omega)\hat{I}_1 \\
\frac{dV}{dt} &= \frac{k}{c}(1-\omega)\hat{I}_1 - \delta_2 V
\end{aligned}
\qquad \textbf{(S. 5)}
$$

And the steady state before treatment is $\bar{\hat{I}}_1 = \frac{\delta_2 c\bar{V}}{k}$ and $\bar{\hat{T}} = \frac{\delta_2 c(\delta_1 + k)}{k}$. Equation (S.5) with $\hat{T}$ replaced by its steady-state value defines a linear system with characteristic equation

$$\pi(\lambda) = \left(-\delta_1 - k(1-\omega) - \lambda\right)\left(-\delta_2 - \lambda\right) - \delta_2(k+\delta_1)(1-\eta)(1-\varepsilon). \qquad \textbf{(S.6)}$$

Defining $\alpha = k(1-\omega)+\delta_1$ and $\theta = \sqrt{(\delta_2 - \alpha)^2 + 4\delta_2(k+\delta_1)(1-\eta)(1-\varepsilon)(1-\omega)}$, we have, solving Eq. (S.6), the eigenvalues $\lambda_{1,2}$= ½ $(\alpha + \delta_2 \pm \theta)$ associated with the eigenvectors:

$$s_1 = \begin{bmatrix} \dfrac{-2c\delta_2(k+\delta_1)(1-\eta)(1-\varepsilon)}{k\left(-\alpha_1 + \delta_2 + \theta\right)} \\ 1 \end{bmatrix}, \quad s_2 = \begin{bmatrix} \dfrac{-2c\delta_2(k+\delta_1)(1-\eta)(1-\varepsilon)}{k\left(-\alpha_1 + \delta_2 - \theta\right)} \\ 1 \end{bmatrix}.$$

Thus, with $V(0) = \bar{V} = V_0$, the solution of the system for $V(t)$ after the start of treatment (t=0) has the form

$$V(t) = \underbrace{\frac{V_0}{2\theta} A e^{-\lambda_1 t}}_{V_1(t)} + \underbrace{\frac{V_0}{2\theta} B e^{-\lambda_2 t}}_{V_2(t)}, \qquad \textbf{(S.7)}$$

where $A=\theta-\alpha-\delta_2+2\delta_2\omega$ and $B=\theta+\alpha+\delta_2-2\delta_2\omega$ thus $A+B=2\theta$, as it should be.

With this solution, we can analyze the profile of viral load decay with and without InSTI. We first analyze under what conditions the A and B terms are positive. For the biologically relevant situation, θ will always be positive, and thus if A and B are both positive, we can observe two early phases of viral decay (phase 1a and 1b), if only one of them is positive, we will only observe one phase of decay (phase 1).

The coefficient $\frac{A}{2\theta}$ will be positive if A>0 or, what is the same, $\theta>\alpha+\delta_2(1-2\omega)$. Defining the total drug effectiveness of a protease inhibitor and reverse transcriptase inhibitor as ξ, such that $(1-\xi) = (1-\varepsilon)(1-\eta)$, we have that if A >0 then

$$\begin{array}{ccc} \theta^2 & > & (\alpha+\delta_2-2\delta_2\omega)^2 \\ (\delta_2-\alpha)^2 + 4\delta_2(\delta_1+k)(1-\xi)(1-\omega) & > & (\alpha+\delta_2)^2 - 4(\alpha+\delta_2)\delta_2\omega + 4\delta_2^2\omega^2 \\ -2\alpha\delta_2 + 4\delta_2(\delta_1+k)(1-\xi)(1-\omega) & > & 2\alpha\delta_2 - 4\alpha\delta_2\omega - 4\delta_2^2\omega(1-\omega) \\ 4\delta_2(\delta_1+k)(1-\xi)(1-\omega) & > & 4\alpha\delta_2(1-\omega) - 4\delta_2^2\omega(1-\omega) \\ 4\delta_2(1-\omega)\left[(\delta_1+k)(1-\xi) - \alpha + \delta_2\omega\right] & > & 0 \\ 4\delta_2(1-\omega)\left[\omega\delta_2 - \left\{\xi\delta_1 + k(\xi-\omega)\right\}\right] & > & 0 \end{array} \qquad \textbf{(S.8)}$$

Therefore, to have the value of $\frac{A}{2\theta}$ greater than zero, we need that $\omega\delta_2>\xi\delta_1+k(\xi-\omega)$ or $\omega > \xi\frac{(\delta_1+k)}{(\delta_2+k)}$. Moreover, as the parameter ω varies between 0 and 1, this condition implies

that $0 \le \xi(\delta_1+k) \le \delta_2+k$. On the other hand, to have $\frac{B}{2\theta}$ greater than zero, we need $\theta > 2\delta_2\omega-(\alpha+\delta_2)$. But since $(\alpha+\delta_2-2\delta_2\omega)^2 = (2\delta_2\omega-\alpha-\delta_2)^2$, from the calculations above, we see that the condition $\omega > \xi\frac{(\delta_1+k)}{(\delta_2+k)}$ is a lower bound on ω to have both, $\frac{A}{2\theta}$ and $\frac{B}{2\theta}$, greater than zero. However, $\frac{B}{2\theta}$ can be greater than zero even if that condition is not fulfilled. For example in the case when the parameter ξ goes to one, the value of $\frac{B}{2\theta}$ has two possible cases depending on the values of δ_2 and α. If $\delta_2>\alpha$, θ will asymptotically go to δ_2-α; and then, $\frac{B}{2\theta} \to \frac{\delta_2-\delta_2\omega}{\delta_2-\alpha}$. In the opposite case, i.e., when $\delta_2<\alpha$, θ will go to α-δ_2, and $\frac{B}{2\theta}$ will go to $\frac{\alpha-\delta_2\omega}{\alpha-\delta_2}$. These expressions are positive for any value of ω in $0 \le \omega < 1$, regardless of the values for the parameters δ_2 and α. Thus, when ξ approaches 1, $B/(2\theta)$ will be positive for any value of ω.

In the scenario when w approaches one, the positivity of $\frac{A}{2\theta}$ and $\frac{B}{2\theta}$ will also depend on the values of δ_2 and α. As before, if $\delta_2>\alpha$, θ will go to δ_2-α; then, the coefficient $\frac{A}{2\theta}$ goes to one and $\frac{B}{2\theta}$ goes to zero. In the opposite case θ approaches α-δ_2; therefore, $\frac{A}{2\theta}$ will go to zero and $\frac{B}{2\theta}$ will go to one. Hence, to have both coefficients $\frac{A}{2\theta}$ and $\frac{B}{2\theta}$ greater than zero, besides the condition presented above as the lower bound on ω, we also need ω to be smaller than one. Otherwise, as the effectiveness ω approaches 1, phases 1a and 1b become just one observable phase.

We can now turn our attention to the two cases of interest, namely therapy with and without the integrase strand transfer inhibitor, RAL.

2.1 Raltegravir-containing therapy

Based on the analysis of the previous section, in the case of RAL-combination therapy with RTIs, i.e., ε=0 and $\xi=\eta$, if $\eta\left(\frac{\delta_1+k}{\delta_2+k}\right) < \omega < 1$ the model predicts two early phases of decay in the viral load, with rates λ_1 and λ_2 (defined after S.6) after initiation of therapy (we call them phases 1a and 1b). Notice that in the case of RAL monotherapy, we have $\xi=\eta=0$. Therefore, the condition to have two phases of decay in the plasma viral load, $0<\omega<1$, is always fulfilled. We notice also that as the effectiveness of RAL in the

combination is close to 1 (but still less than 1), the viral load decay rates λ_1 and λ_2 approach δ_2 and δ_1, respectively. Therefore, if the effectiveness of RAL is very high, the model predicts that phase 1b of the viral load will be due to the loss of infected cells without integrated provirus at rate δ_1.

2.2 Therapy without raltegravir (*i.e.*, just RTI and PI)

In the case of an RTI-PI combination regimen without InSTI, *i.e.*, ω=0, the coefficients A and B are $A=\theta-\alpha-\delta_2$ and $B=\theta+\alpha+\delta_2$. In this case B is always positive, but A is positive only if $\theta>a+d_2$. Using the same procedure as in (S.8), $\theta>\alpha+\delta_2$ if and only if $\xi(\delta_1+k)<0$. However, the parameters ξ, k and δ_1 are always positive, which implies that A is always negative. Notice, however, that even though B is always positive, θ has two asymptotic values as ξ approaches one. If $\delta_2>(\delta_1+k)$, θ will asymptotically go to $\delta_2-(d_1+k)$ as ξ goes to one. In the opposite case θ will go $(\delta_1+k)-\delta_2$. Therefore, $\frac{B}{2\theta}\rightarrow\frac{\delta_2}{\delta_2-(\delta_1+k)}$ and $\lambda_2\rightarrow(\delta_1+k)$ if $\delta_2>(\delta_1+k)$, or $\frac{B}{2\theta}\rightarrow\frac{(\delta_1+k)}{(\delta_1+k)-\delta_2}$ with $\lambda_2\rightarrow\delta_2$ in the opposite case, giving two possible scenarios for the rate of decay of the viral load during the first phase in the absence of RAL. That is, in the absence of RAL, the first phase decay rate could correspond to the loss of pre-integration infected cells or to the loss of productively infected cells, respectively. In any case, in the absence of raltegravir, we can't observe an early second phase in the kinetics of viral load decline unless there is another source of virus, such as long-lived infected cells. Thus, phase 1b can only be seen in RAL-based regimens.

3 Mathematical model with long-lived cells

We now study the full initial model (Eq. S.3) with long-lived cells, which can generate the long-term slower decay (phase 2) observed in the data. We are interested in finding conditions to observe 2 or 3 phases of decay during the first 30 days of treatment.

Using the steady state values for $\hat{T}$ and $\hat{M}$, and using the fraction of virus produced by short-lived cells and long-lived cell, $f_I=\frac{\bar{V}_I}{\bar{V}}$ and $f_M=\frac{\bar{V}_M}{\bar{V}}$, respectively, the characteristic equation of the linear system in Eq. (S.3) has the form

$$\begin{aligned}\pi(\lambda) \;=\; & (-\alpha_1-\lambda)(-\alpha_2-\lambda)(-\delta_2-\lambda)(-\delta_{M2}-\lambda)\\ & -(1-\eta)(1-\varepsilon)(1-\omega)(-\alpha_1-\lambda)(-\delta_2-\lambda)(k_1+\delta_{M1})f_M\delta_{M2}\quad,\\ & -(1-\eta)(1-\varepsilon)(1-\omega)(-\alpha_2-\lambda)(-\delta_{M2}-\lambda)(k+\delta_1)f_I\delta_2\end{aligned}\qquad \textbf{(S.9)}$$

where $\alpha_1=\delta_1+k(1-\omega)$ and $\alpha_2=\delta_{M1}+k_1(1-\omega)$. Since the parameter f_M should be very small (*i.e.*, the second phase is only observed after the viral load has decayed more than 1 $\log_{10}$) we may assume that $(-\alpha_2-\lambda)(-\delta_{M2}-\lambda)(\delta_{M1}+k)f_I\delta_2>>(-\alpha_1-\lambda)(-\delta_2-\lambda)(\delta_1+k_1)f_M\delta_{M2}$. Using this assumption, we obtain an approximation of the characteristic equation (S.9) with the

form: $\pi(\lambda) \approx (-\alpha_1 - \lambda)(-\alpha_2 - \lambda)(-\delta_2 - \lambda)(-\delta_{M2} - \lambda) - (1-\eta)(1-\varepsilon)(1-\omega)(-\alpha_2 - \lambda)(-\delta_{M2} - \lambda)(k+\delta_1) f_I \delta_2$. This can be solved to obtain the following eigenvalues of the system in Eq. (S.3),

$$\begin{aligned} \lambda_1 &= \frac{\alpha_1 + \delta_2 + \theta_f}{2} \\ \lambda_2 &= \frac{\alpha_1 + \delta_2 - \theta_f}{2} \\ \lambda_3 &= \delta_{M2} \\ \lambda_4 &= \alpha_2 \end{aligned} \qquad \textbf{(S.10)}$$

With $\theta_f = \sqrt{(\delta_2 - \alpha_1)^2 + 4k\frac{\bar{\hat{T}}}{c}(1-\eta)(1-\varepsilon)(1-\omega) f_I}$. These eigenvalues are associated with the eigenvectors,

$$s_1 = \begin{bmatrix} \frac{-2\bar{\hat{T}}(1-\eta)(1-\varepsilon)}{-\alpha_1+\delta_2+\theta_f} \\ 0 \\ 1 \\ 0 \end{bmatrix}, \quad s_2 = \begin{bmatrix} \frac{-2\bar{\hat{T}}(1-\eta)(1-\varepsilon)}{-\alpha_1+\delta_2-\theta_f} \\ 0 \\ 1 \\ 0 \end{bmatrix}, \quad s_3 = \begin{bmatrix} \frac{\bar{\hat{T}}(1-\eta)(1-\varepsilon)(\delta_2-\delta_{M2})}{-k\frac{\bar{\hat{T}}}{c}(1-\eta)(1-\varepsilon)(1-\omega)+(\alpha_1-\delta_{M2})(\delta_2-\delta_{M2})} \\ 0 \\ \frac{k\frac{\bar{\hat{T}}}{c}(1-\eta)(1-\varepsilon)(1-\omega)}{-k\frac{\bar{\hat{T}}}{c}(1-\eta)(1-\varepsilon)(1-\omega)+(\alpha_1-\delta_{M2})(\delta_2-\delta_{M2})} \\ 1 \end{bmatrix}, \quad s_4 = \begin{bmatrix} \frac{\bar{\hat{T}}(1-\eta)(1-\varepsilon)(\alpha_2-\delta_2)}{-k\frac{\bar{\hat{T}}}{c}(1-\eta)(1-\varepsilon)(1-\omega)+(\alpha_1-\alpha_2)(\alpha_2-\delta_2)} \\ -\frac{c(\alpha_2-\delta_{M2})}{k(1-\omega)} \\ \frac{k\frac{\bar{\hat{T}}}{c}(1-\eta)(1-\varepsilon)(1-\omega)}{-k\frac{\bar{\hat{T}}}{c}(1-\eta)(1-\varepsilon)(1-\omega)+(\alpha_1-\alpha_2)(\alpha_2-\delta_2)} \\ 1 \end{bmatrix}$$

Thus, with $\bar{V}_M + \bar{V}_I = V_M(0) + V_I(0) = V_0$, the approximate solution for $V(t)=V_I(t)+V_M(t)$ in Eq. (S.3) has the form,

$$V(t) \approx \underbrace{V_0 C_1 e^{-\lambda_1 t}}_{V_1(t)} + \underbrace{V_0 C_2 e^{-\lambda_2 t}}_{V_2(t)} + \underbrace{V_0 C_3 e^{-\lambda_3 t}}_{V_3(t)} + \underbrace{V_0 C_4 e^{-\lambda_4 t}}_{V_4(t)} , \qquad \textbf{(S.11)}$$

with coefficients,

$$\begin{aligned} C_1 &= 1 - (C_2 + C_3 + C_4), \\ C_2 &= \frac{\alpha_1 - \delta_2 + \theta_f}{2\theta_f} \times \\ &\begin{bmatrix} f_I - \frac{\alpha_1 - \delta_2 + \theta_f}{2(\delta_1 + k)(1-\eta)(1-\varepsilon)} - \frac{f_M\left[(\delta_2 - \delta_{M2})(\alpha_1 - \delta_2 - \theta_f) - 2\delta_2(\delta_1 + k) f_I (1-\eta)(1-\varepsilon)(1-\omega)\right]}{2\left[(\alpha_1 - \delta_{M2})(\delta_2 - \delta_{M2}) + \delta_2(\delta_1 + k) f_I (1-\eta)(1-\varepsilon)(1-\omega)\right]} \\ -\frac{\delta_2 f_M (1-\omega)\left[(\delta_2 - \alpha_2)(\delta_2 - \delta_{M2})(\alpha_1 - \delta_2 - \theta_f) - 2\delta_2(\delta_1 + k) f_I (1-\eta)(1-\varepsilon)(1-\omega)(\alpha_1 - 2\alpha_2 + 3\delta_2 - 2\delta_{M2} + \theta_f)\right]}{2\left[(\alpha_1 - \alpha_2)(\delta_2 - \alpha_2)(\alpha_1 - \delta_{M2})(\delta_2 - \delta_{M2}) + \left[\delta_2(\delta_1 + k) f_I (1-\eta)(1-\varepsilon)(1-\omega)\right]^2\right]} \end{bmatrix}, \qquad \textbf{(S.12)} \\ C_3 &= f_M \frac{(\alpha_1 - \delta_{M2})(\alpha_2 - \omega\delta_{M2})(\delta_2 - \delta_{M2})}{(\alpha_2 - \delta_{M2})\left[(\alpha_1 - \delta_{M2})(\delta_2 - \delta_{M2}) - \delta_2(\delta_1 + k) f_I (1-\eta)(1-\varepsilon)(1-\omega)\right]}, \\ C_4 &= f_M \frac{\delta_{M2}(\alpha_1 - \alpha_2)(\delta_2 - \alpha_2)(1-\omega)}{(\delta_{M2} - \alpha_2)\left[(\alpha_1 - \alpha_2)(\delta_2 - \alpha_2) - \delta_2(\delta_1 + k) f_I (1-\eta)(1-\varepsilon)(1-\omega)\right]}. \end{aligned}$$

We found numerically that $V_1(t)$ and $V_2(t)$ in Eq. (S.11) have approximately the same decay pattern as predicted for the corresponding components in Eq. (S.7).

To analyze different scenarios for the virus produced by the long-lived cells, we assume that ε and η are close to one. In this case, the coefficients C_3 and C_4 approach asymptotically $f_M \frac{\alpha_2 - \omega\delta_{M2}}{\alpha_2 - \delta_{M2}}$ and $f_M \frac{\delta_{M2}(1-\omega)}{\delta_{M2} - \alpha_2}$, respectively. Notice that since $V_M(0) = V_0 f_M$, the asymptotic values of C_3 and C_4 are proportional to the total contribution of long-lived infected cells to viral load, $V_M(0)$. In the presence of RAL, i.e., $\omega>0$, if $\alpha_2>\delta_{M2}$, the asymptotic value for C_3 is positive and C_4 is negative. On the other hand, if $\alpha_2<\delta_{M2}$, then C_4 is positive and C_3 is positive if $\omega > \frac{\delta_{M1}+k_1}{\delta_{M2}+k_1}$. Thus in the presence of RAL if $\delta_{M1}+k_1(1-\omega)>\delta_{M2}$ three phases of decay are possible: the first two phases with the same rates as in the simplified model without long-lived cells (S.7 above); and the third phase with a decay rate of δ_{M2}. On the other hand, if $\delta_{M1}+k_1(1-\omega)<\omega\delta_{M2}$ four phases of decay are possible, where the first two are the same as before, and the other two have rates $\delta_{M1}+k_1(1-\omega)$ and δ_{M2}. Notice, however, that in this case, if $\delta_{M2}=\delta_2$, only three phases of decay are seen, with the last decay rate equal to $\delta_{M1}+k_1(1-\omega)$ (see next section for details).

In the absence of RAL, *i.e.*, when $\omega=0$, C_1 is always negative and C_2 is positive (equivalent to $\frac{B}{2\theta}$ in section 1.2). This means that there is only a phase 1, which is not subdivided into phase 1a and 1b. In addition, for $\omega=0$, only one of the coefficients C_3 or C_4 is greater than zero, and we will have only one phase of decay later in treatment. If $\delta_{M1}+k_1>\delta_{M2}$, then C_3 is positive and C_4 is negative, and the second phase decay rate is δ_{M2}. If $\delta_{M1}+k_1<\delta_{M2}$, then C_3 is negative and C_4 is positive, and the second phase decay rate is $\delta_{M1}+k_1$. Thus, in the absence of RAL only two phases of decay will be seen over the first 30 days of treatment.

3.1 Model assuming $\delta_{M2} = \delta_2$

The results of our simulations and fits (see main text and section 4.4 below), indicated that a scenario with $\delta_{M2} = \delta_2$ is the most parsimonious to explain the data. In this case, we can add the equations for $V_I(t)$ and $V_M(t)$ in Eq. (S.3), obtaining the following system of differential equations

$$\begin{aligned}
\frac{d\hat{I}_1}{dt} &= (1-\eta)(1-\varepsilon)\hat{T}V - \delta_1\hat{I}_1 - k(1-\omega)\hat{I}_1 \\
\frac{d\hat{M}_1}{dt} &= (1-\eta)(1-\varepsilon)\hat{M}V - \delta_{M1}\hat{M}_1 - k_1(1-\omega)\hat{M}_1 \\
\frac{dV}{dt} &= \frac{k}{c}(1-\omega)\hat{I}_1 + \frac{k_1}{c}(1-\omega)\hat{M}_1 - \delta_2 V
\end{aligned} \qquad \textbf{(S.13)}$$

One still expects that before therapy, the contribution to viral load from the long-lived infected cells ($\hat{M}_1$) is a small fraction of the total virus. Indeed, at steady state before therapy $\frac{\bar{V}_M}{\bar{V}_I} = \frac{k_1\bar{\hat{M}}_1}{k\bar{\hat{I}}_1}$, and as before $f_I = \frac{\bar{V}_I}{\bar{V}}$ and $f_M = \frac{\bar{V}_M}{\bar{V}}$. We can then calculate the relative

fraction of infection events leading to short-lived productively infected cells as $\frac{\bar{\bar{T}}\bar{V}}{\bar{\bar{T}}\bar{V}+\bar{\hat{M}}\bar{V}}=\frac{\delta_1+k}{\delta_1+k+(\delta_{M_1}+k_1)\frac{kf_M}{k_1 f_I}}$, and the fraction of infection events leading to long-lived infected cells as $1-\frac{\bar{\hat{T}}\bar{V}}{\bar{\hat{T}}\bar{V}+\bar{\hat{M}}\bar{V}}$.

We now solve the system (S.13). With V_0 the initial value of the viral load, and using the same assumptions and procedure of the previous section, the solution for $V(t)$ in equation (S.13) has the form,

$$V(t)\approx V_0\left[C_{1a}e^{-\frac{(\alpha_1+\delta_2-\theta_f)}{2}t}+C_{1b}e^{-\frac{(\alpha_1+\delta_2+\theta_f)}{2}t}+C_2 e^{-[\delta_{M1}+k_1(1-\omega)]t}\right],$$

$$C_{1a}=\frac{\alpha_1-\delta_2+\theta_f}{2\theta_f}\left[1-\frac{\delta_2(1-f_I)(1-\omega)(\alpha_1-2\alpha_2+\delta_2+\theta_f)}{2(\alpha_1-\alpha_2)(\delta_2-\alpha_2)-2\delta_2(\delta_1+k)f_I(1-\eta)(1-\varepsilon)(1-\omega)}-\frac{\alpha_1-\delta_2-\theta_f}{2(\delta_1+k)(1-\eta)(1-\varepsilon)}\right], \quad \textbf{(S.14)}$$

$$C_{1b}=1-C_{1a}-C_2,$$

$$C_2=(1-f_I)\frac{\delta_2(\alpha_1-\alpha_2)(1-\omega)}{(\alpha_1-\alpha_2)(\delta_2-\alpha_2)-\delta_2(\delta_1+k)f_I(1-\eta)(1-\varepsilon)(1-\omega)}.$$

In the case of InSTI-free therapy (w=0) we have that C_{1b} is negative. In this scenario the first phase decay rate depends on the values of δ_2 and α_1. Phase two begins when the viral load, $V(t)$, goes below V_0C_2 and its decay rate is $\delta_{M1}+k_1(1-\omega)$. Finally, we define this transition time as the time t_{12} when $C_{1a}e^{-\frac{(\alpha_1+\delta_2-\theta_f)}{2}t_{12}}=C_2 e^{-[\delta_{M1}+k_1(1-\omega)]t_{12}}$, which leads to

$$t_{12}=\frac{\log(C_{1a|\{\omega=0\}})-\log(C_{2|\{\omega=0\}})}{\frac{(\delta_1+k+\delta_2-\theta_f)}{2}-(\delta_{M1}+k_1)}. \quad \textbf{(S.15)}$$

3.2 Model assuming $\delta_{M2}=\delta_2$ and $\eta=\varepsilon=1$

To gain more insight into the model defined in S.13, we now assume $\eta=\varepsilon=1$, that is perfect efficacy of the non-InSTI drugs (the corresponding fits under this assumption did not present significant differences with the fits presented in the main text. Compare Table C and D). The model in equation (S.13) is simplified to the following ordinary differential equation system: $\frac{d\hat{I}_1}{dt}=-\delta_1\hat{I}_1-k(1-\omega)\hat{I}_1$, $\frac{d\hat{M}_1}{dt}=-\delta_{M1}\hat{M}_1-k_1(1-\omega)\hat{M}_1$ and $\frac{dV}{dt}=\frac{k}{c}(1-\omega)\hat{I}_1+\frac{k_1}{c}(1-\omega)\hat{M}_1-\delta_2 V$. With V_0 the initial value of the viral load when the treatment starts, the exact solution for $V(t)$ has the form,

$$V(t)=V_0\left[C_{1a}e^{-\delta_2 t}+C_{1b}e^{-[\delta_1+k(1-\omega)]t}+C_2 e^{-[\delta_{M1}+k_1(1-\omega)]t}\right],$$
$$C_{1a}=1-C_{1b}-C_2,$$
$$C_{1b}=f_I\frac{\delta_2(1-\omega)}{\delta_2-\left[\delta_1+k(1-\omega)\right]}, \qquad \textbf{(S.16)}$$
$$C_2=(1-f_I)\frac{\delta_2(1-\omega)}{\delta_2-\left[\delta_{M1}+k_1(1-\omega)\right]}.$$

The coefficients C_{1a}, C_{1b} and C_2 represent the fraction of viral load at time 0 for phases 1a, 1b and 2, coming from short-lived cells after integration, and short- and long-lived cells before integration, respectively. The rates of decay of these phases are δ_2, $\delta_1+k(1-\omega)$ and $\delta_{M1}+k_1(1-\omega)$ day^{-1}, respectively. We define the transition from one phase to the next as occurring when the viral load of the two phases are equal, that is the transition from phase 1a to phase 1b occurs when the viral load due to the decay of C_{1a} is the same as the viral load due to the decay of C_{1b}.

The dynamics of the viral load second phase is defined by the term $(1-f_I)\sigma e^{-[\delta_{M1}+k_1(1-\omega)]t}$ with $\sigma=\frac{\delta_2(1-\omega)}{\delta_2-\left[\delta_{M1}+k_1(1-\omega)\right]}$ according to equation S.16. With this model the ratio of the viral load in the combination treatment (with RAL) to the viral load in the quad treatment (without RAL) during the second phase has the form $\frac{\sigma_{\omega>0}e^{-[\delta_{M1}+k_1(1-\omega)]t}}{\sigma_{\omega=0}e^{-[\delta_{M1}+k_1]t}}$. In the case when $\delta_2 \gg \delta_{M1}+k_1$, we can simplify σ, and this ratio is simply $(1-\omega)e^{\omega k_1 t}$.

4 Fitting Procedure

Before fitting the data with the models described above, we explored the range of possible values for the parameters by simulation.

4.1 Initial Simulations

We first explored the possible range of values for the new parameters in our model, δ_{M1}, δ_{M2} and k_1, keeping fixed the remaining parameters based on estimates from previous studies[1,7,8]. Specifically we performed 64,000 (40 values for each parameter) simulations of the model in equation (S.3) for different values of these parameters. We looked for the number of cases out of those 64,000 where the viral load decay under RAL-based and RAL-free regimes fulfilled the following criteria (assuming that long-lived cells pre-integration die slower than when they become productively infected, *i.e.*, $\delta_{M1}<\delta_{M2}$): (1) the viral load level at the start of the second phase under RAL is decreased by 70%-95% in relation to the corresponding viral load without RAL; (2) the ratio of slopes of the second phase of the viral load under RAL over the case without RAL is in the range 0.5-1; (3) slopes of the second phase in both treatment scenarios are between 0.02 day^{-1} and 0.1 day^{-1}; and (4) the viral load at 30 days is greater than 10 copies/mL for any type of treatment. These four criteria formalize the observations made by us and others regarding the decay of viral load in the presence of RAL. The fixed parameter values used were: $V_I(0)/V(0)=0.98$, k=2.6 day^{-1}, c=23 day^{-1}, δ_1=0.15 day^{-1} and $V(0)$= 4.6 based on previous

studies[1,7,8]. For RAL-combination treatment we used η=0.95, ε=0 and ω=0.94, and for quad-based treatment η=ε=0.95 and ω=0. We simulated 40 different values each of k_1, δ_{M1} and δ_{M2} in the ranges (0.005-1 day^{-1}), (0.01-0.15 day^{-1}) and (0.04-1 day^{-1}), respectively. We found that these conditions are only satisfied for small values of δ_{M1} (<0.07 day^{-1}) and k_1 (<0.08 day^{-1}), along with values of δ_{M2} greater than 0.15 day^{-1} (Figure A). These results gave us starting guesses for these parameters in the viral load fits.

4.2 Fixed Parameters

As we have discussed before[1], the viral load decay kinetics are not sensitive to the actual values of η and ε, when these are greater than ~90% (see section 4.4). Therefore, we kept the efficacy of RTIs and PIs in the model fixed at η=ε=0.95. To further simplify the fitting procedure and obtain convergence, we also fixed the efficacy of RAL at their estimated population values in our previous study, *i.e.*, ω=0.94 for RAL-combination therapy and ω=0.997 for RAL-monotherapy[1]; and used a value of 2.6 day^{-1} for the integration rate, k, as before[1]. In our initial analyses of the two phases of decay in the quad-regimen, using a two-exponential function, we found that the coefficient for the first phase was 0.98×V(0). This should correspond to the fraction of total virus produced from activated-short-lived infected cells, $V_I(0)/V(0)$, and we used this value as an estimate of this initial condition. Finally, we fixed the value of virus clearance, c, at 23 day^{-1} (8).

4.3 Fitting details

We were interested in the case where M_1 and M_2 represent long-lived cells with loss rates smaller than those for I_1 and I_2. In addition, one expects that productively infected cells are lost faster than infected cells with unintegrated viral DNA. Therefore, we used initial guesses for the fitting procedure such that $\delta_{M1}<\delta_{M2}<\delta_1<\delta_2$. In addition, Sedaghat *et. al.* suggested based on theoretical analysis of this type of model that $\delta_{M1}+k_1>\delta_{M2}$ [4,6]. Putting all this together, we used as initial guesses of the fitting procedure δ_{M2}= 0.05 day^{-1}, assuming it corresponds to the slope of the second phase in the viral load decline [3], δ_{M1} = 0.03 day^{-1}, so that $\delta_{M1}<\delta_{M2}$ and k_1 =2.6 day^{-1}, assuming the integration rate was similar in short- and long-lived infected cells. For the parameters δ_1 and δ_2 we used as initial guesses our previous estimates of 0.15 day^{-1} [1] and 1 day^{-1} [7]. In all cases we used a value of 0.01 as the initial guess for the variance of the random effects.

The estimation procedure converged to one of two different scenarios, either $\delta_{M2}>\delta_1$ or $\delta_{M2}<\delta_1$ (see two first rows of Table A). Surprisingly, the case with $\delta_{M2}>\delta_1$ had better statistical support (ΔAIC=19.3 Table A), contrary to our initial assumption used for the starting guess of the fitting procedure. In addition, in this case the estimate of δ_1 was similar to our previous estimate analyzing the early viral load decay until day 10 [1]. Moreover we found that this case, also contrary to our initial assumptions, resulted in $\delta_{M1}+k_1<\delta_{M2}$ (Table A), which implies that the model predicts three phases of viral decay (1a, 1b and 2), with a phase 2 rate of $\delta_{M1}+k_1(1-\omega)$. Still, it was difficult to estimate both δ_{M1} and k_1, since when one increased the other tended to decrease. Therefore, we next performed the fitting procedure for different fixed values of δ_{M1}.

Based on these initial results, we re-did the fits fixing δ_{M1} with values equal to 0.1, 0.05, 0.02, 0.01 and 0.005 day^{-1}. The best fits were obtained with δ_{M1}=0.01 day^{-1} or δ_{M1}=0.02

day^{-1} based on AIC (see Table B). The value of k_1 estimated for all cases was small compared to k, with values smaller than 0.05 day^{-1}. These estimates are consistent with the results of our exploration of parameter space by simulation (see above). Finally, the estimate of δ_{M2} for the best model was ~0.90 day^{-1}, very close to the estimated value of δ_2=0.86 day^{-1}. Thus, we refit the data with the hypothesis that the "long-lived cells" may have a slow decay/integration in the pre-integration state (M_1), but after becoming productively infected (M_2), they are lost at rates equal to activated-short-lived cells, *i.e.*, assuming $\delta_{M2}=\delta_2$ (see section 3.1 above). Under this assumption, again either δ_{M1}=0.01 or 0.02 day^{-1} fit the data best (see Table C). In any case, the population estimates for δ_1 and δ_2 are 0.24 day^{-1} and 0.85 day^{-1}, respectively. As expected, the estimate of the rate of integration for the "long-lived cells", k_1, depended on the value of δ_{M1}. For the case of δ_{M1}= 0.01 day^{-1}, k_1 was estimated to be 0.037 day^{-1}, and for δ_{M1}= 0.02 day^{-1} k_1 was estimated as 0.017 day^{-1}. These values correspond to a pre-integration half-life for long-lived cells between ~15 and ~19 days, respectively (See Figs. B-D). We also tested in our mixed effects model if the value of k_1 depended on the type of treatment (RAL-free vs. RAL-containing) and found that it was not significantly different (p>0.26). We performed the same fitting procedure assuming 100% efficacy for RTIs and PIs (see section 3.2), and obtained very similar results (Table D).

4.4 Testing additional assumptions

We further tested three assumptions made in our analyses. First, we assumed that the efficacy of RTIs and PIs were high, with values of η and ε equal to 0.95. Second, we assumed that the efficacies of the drug regimen in blocking specific steps of the viral lifecycle were the same in short-lived (I_1) and long-lived (M_1) cells. Third, we assumed that the level of target cells remains constant at approximately the pre-therapy steady state during the time of analyses (~30 days).

With respect to the first issue, we performed simulations of the model in equation S.13 using the best estimates of the previous section but using lower values of η and ε equal to 0.9 and 0.8. These changes resulted in minor differences in the viral load profiles, which are hardly noticeable (Fig. F).

Regarding the second issue, we performed simulations of the following generalized model of equation S.3, with different drug efficacy for reverse transcriptase and integrase inhibitors for cells with short (η, ω) or long integration (η_1, ω_1):

$$\begin{aligned}
\frac{d\hat{I}_1}{dt} &= (1-\eta)(1-\varepsilon)\hat{T}V - \delta_1\hat{I}_1 - k(1-\omega)\hat{I}_1 \\
\frac{d\hat{M}_1}{dt} &= (1-\eta_1)(1-\varepsilon)\hat{M}V - \delta_{M1}\hat{M}_1 - k_1(1-\omega_1)\hat{M}_1 \\
\frac{dV_I}{dt} &= \frac{k}{c}(1-\omega)\hat{I}_1 - \delta_2 V_I \\
\frac{dV_M}{dt} &= \frac{k_1}{c}(1-\omega_1)\hat{M}_1 - \delta_{M2}V_M
\end{aligned} \quad \textbf{(S.17)}$$

We used the best estimates of the previous section keeping η=0.95 but changing the value of η_1 to 0.9 and 0.85. Fig. G(a) shows that the viral profile is not significantly different by

changing η_1. We also repeated the procedure presented in section 4.1, to explore the parameter space by simulation under biologically observed constraints, but using equation S.17 and narrowing the set of values for the reduction of the second phase by raltegravir between 85-95% since the reduction of the viral load second phase is about 90% in patients under RAL compared to those under treatment without RAL, fixing ω=0.94 and scanning the value of ω_1 from 0.5 to 1. The purpose of the simulation was to see if it was possible to obtain values of δ_{M2} smaller than $\delta_{M1}+k_1$ for some values of w_1 for the criteria defined in section 4.1, i.e. if it was possible to find a different explanation for the rates of viral decay than the one presented in the main text. Fig. G(b) shows that that even allowing ω_1 to vary, the values of δ_{M2} have to be greater than $\delta_{M1}+k_1$ as obtained by our original analysis. Interestingly, the mode of ω_1 was 0.93 as depicted in Fig. G(b), indicating that our assumption of $\omega\sim\omega_1$ is good.

In relation to the third issue, we used a generalized model of equation S.1 including the dynamics of target cells, T and M. We assume target cells T are produced at constant rate λ die at rate d, and become infected at rate β. We assume target cells M have the same dynamics but with production and infection rates λ_1 and β_1. For simplicity, we assumed the same d, but this has little influence on the dynamics over the short term studied. Therefore, we included the following two equations in the model presented in equation (S.1):

$$\begin{aligned}\frac{dT}{dt} &= \lambda - dT - (1-\eta)\beta T V_i \\ \frac{dM}{dt} &= \lambda_1 - dM - (1-\eta)\beta_1 M V_i\end{aligned} \qquad \textbf{(S.18)}$$

Redefining variables as before, so that $\hat{T} = p\beta T$, $\hat{M} = p_1\beta_1 M$, $\hat{\lambda} = p\beta\lambda$ and $\hat{\lambda}_1 = p_1\beta_1\lambda_1$, we can re-write model S.13 adding two equations for the target cells, obtaining the following system:

$$\begin{aligned}\frac{d\hat{T}}{dt} &= \hat{\lambda} - d\hat{T} - (1-\eta)(1-\varepsilon)\beta\hat{T}V_i \\ \frac{d\hat{M}}{dt} &= \hat{\lambda}_1 - d\hat{M} - (1-\eta)(1-\varepsilon)\beta_1\hat{M}V_i \\ \frac{d\hat{I}_1}{dt} &= (1-\eta)(1-\varepsilon)\hat{T}V - \delta_1\hat{I}_1 - k(1-\omega)\hat{I}_1 \\ \frac{d\hat{M}_1}{dt} &= (1-\eta)(1-\varepsilon)\hat{M}V - \delta_{M1}\hat{M}_1 - k_1(1-\omega)\hat{M}_1 \\ \frac{dV}{dt} &= \frac{k}{c}(1-\omega)\hat{I}_1 + \frac{k_1}{c}(1-\omega)\hat{M}_1 - \delta_2 V\end{aligned} \qquad \textbf{(S.19)}$$

We simulated equations (S.19) with the values of the best fit in Table C, using the steady state in the absence of treatment as initial conditions, and choosing values for d equal to 0.1, 0.01 and 0.001 day^{-1} and values for β and β_1 equal to 10^{-8}, 10^{-7}, 10^{-6} and 10^{-5} ml day^{-1}. In Fig. H, we plot the comparison of the viral load profiles predicted by model S.13, assuming constant target cells, with respect to the model in equation S.19, assuming variable target cells. The figure clearly shows that for the 48 cases (3 values of d × 4

values of $\beta \times 4$ values of β) simulated there is no significant difference in the viral load profiles by assuming constant (solid lines) or variable (dashed lines) target cells during the initial 50 days of treatment.

Each of the assumptions presented above are commonly made in analyzing viral dynamics, and our results here justify that indeed they do not lead to relevant changes in our fitting results.

5 Supplementary Figures

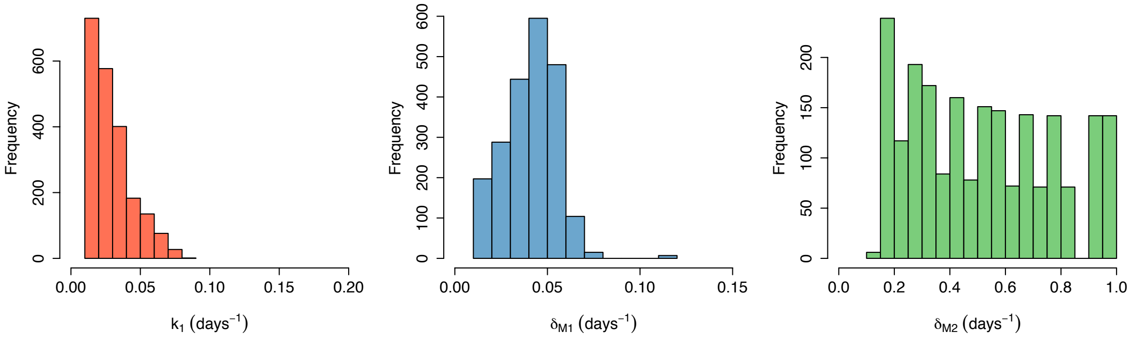

Figure A. Distributions of the values of k_1, δ_{M1} and δ_{M2} where the viral load under RAL-based and RAL-free regimes satisfy biological criteria. These criteria are (assuming $\delta_{M1} < \delta_{M2}$): (1) the second phase under RAL starts at a viral load level that is lower than the start of the second phase in RAL-free regimens by 70%-95%; (2) the ratio of slopes for the second phase decay of the viral load under RAL over the case without RAL is in the range 0.5-1; (3) slopes of the second phase in both treatment scenarios are in the range 0.02 day^{-1} to 0.1 day^{-1}; and (4) the viral load at 30 days is greater than 10 copies/mL for any type of treatment. We fixed the values of the following parameters: $V_I(0)/V(0) = 0.98$, k=2.6 day^{-1}, c=23 day^{-1} δ_1=0.15 day^{-1} and $V(0) = 4.6$ based on previous studies [1,7,8]. For RAL-combination treatment we used η=0.95, ε=0 and ω=0.94, and for quad-based treatment η=ε=0.95 and ω=0. We simulated for 40 different values each of k_1, δ_{M1} and δ_{M2} in the ranges (0.01-1 day^{-1}), (0.005-0.15 day^{-1}) and (0.04-1 day^{-1}), respectively, for a total of 64,000 parameter sets.

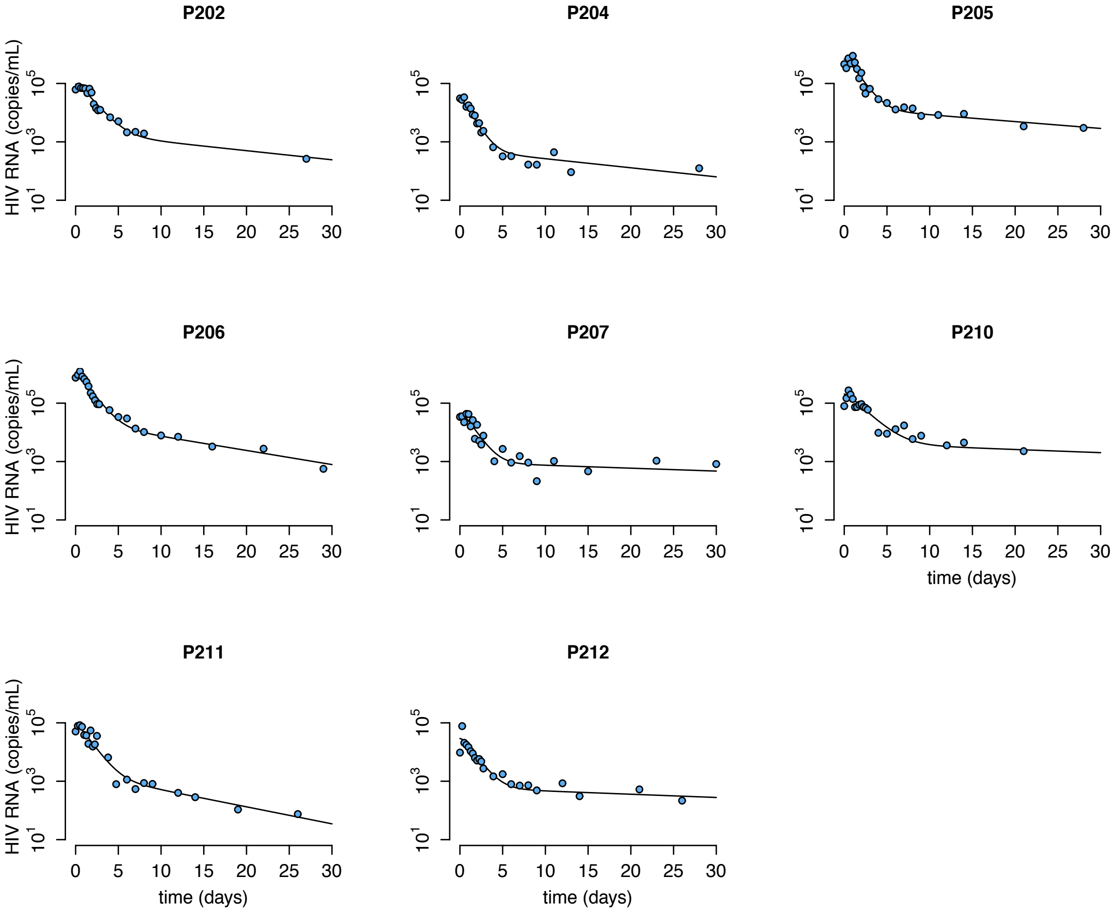

Figure B. Individual fits for the quad-based therapy data assuming δ_2=δ_{M2} and δ_{M1}=0.02 per day in equation (S.3) (rewritten in equation S.13). Circles represent HIV-RNA measurements; solid lines represent best fits from model.

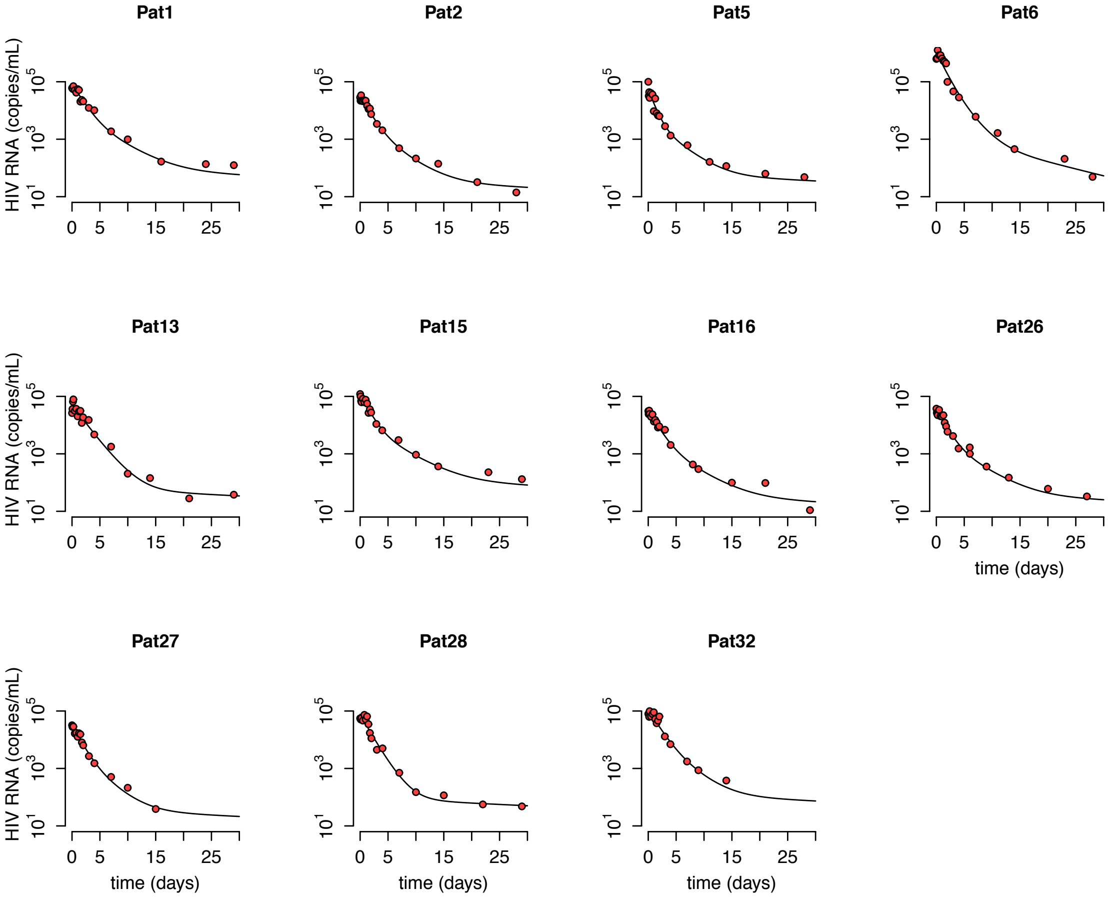

Figure C. Individual fits to the ACTG5249s-RAL-combination therapy data assuming $\delta_2=\delta_{M2}$ and $\delta_{M1}=0.02$ per day in equation (S.3) (rewritten in equation S.13). Circles represent HIV-RNA measurements, solid lines represent best fits from model.

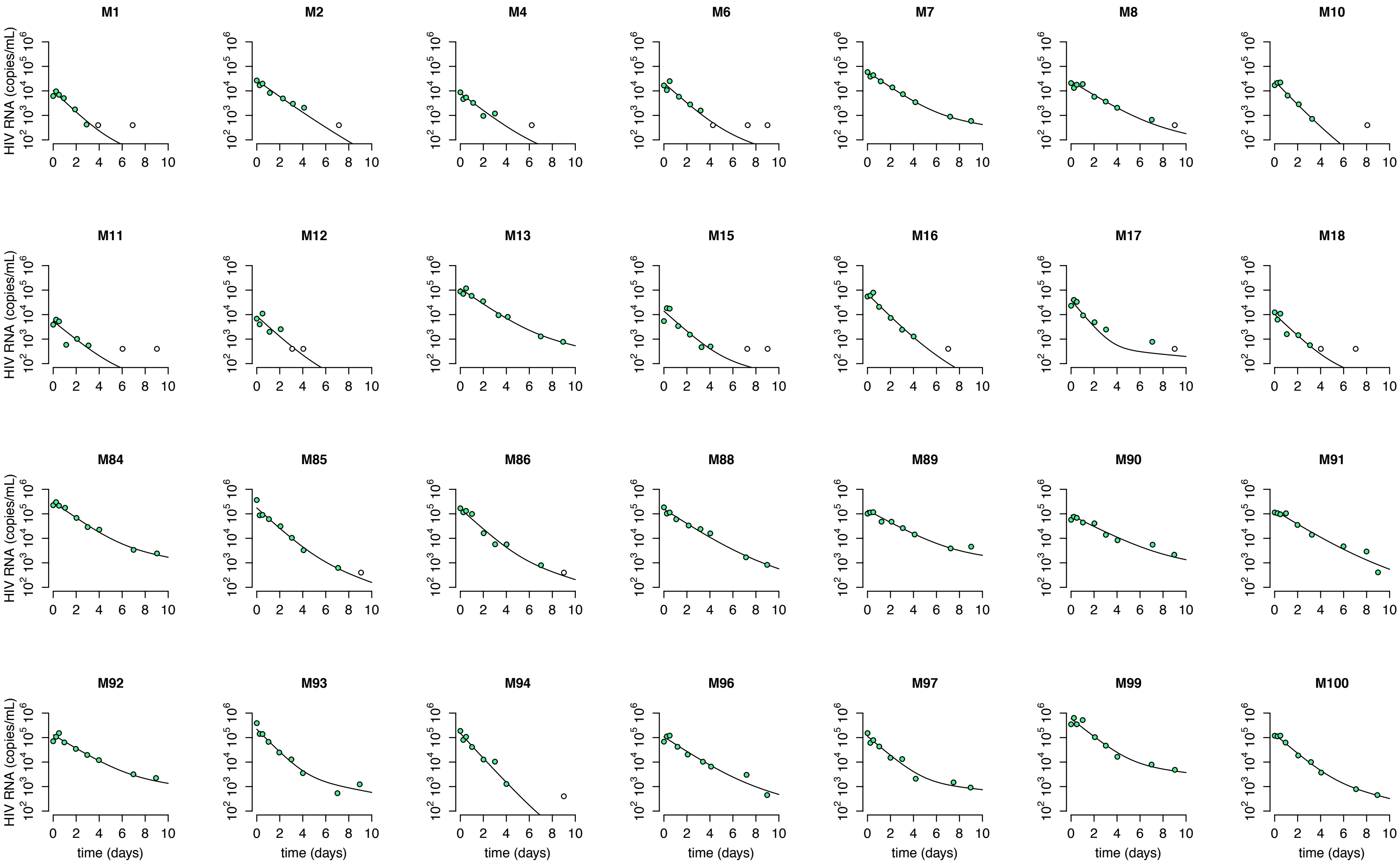

Figure D. Individual fits to RAL monotherapy data assuming $\delta_2=\delta_{M2}$ and $\delta_{M1}=0.01$ per day per day in equation (S.3) (rewritten in

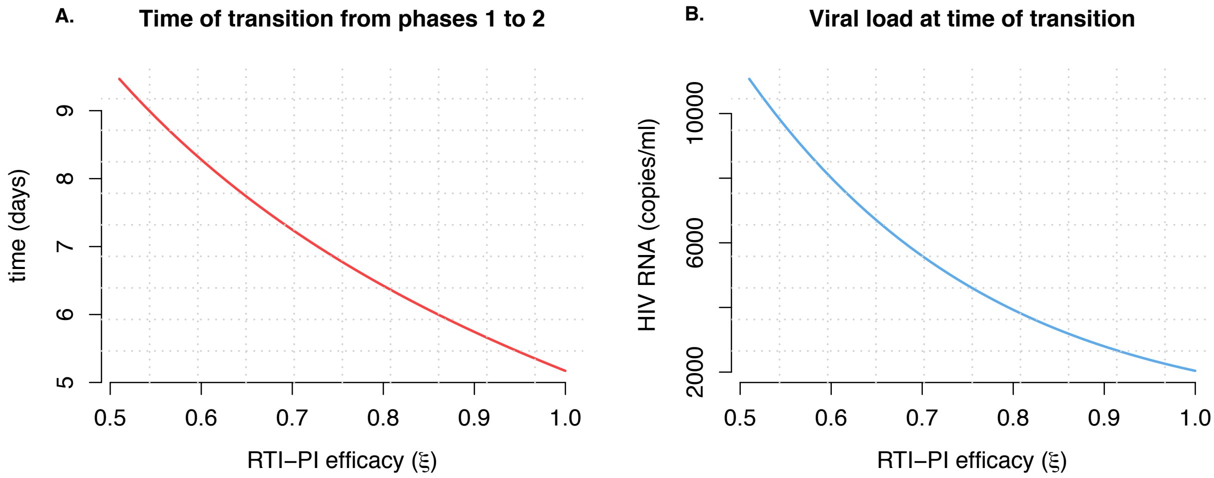

Figure E. Transition between phase 1 and phase 2 in the quad-therapy data. As the combined efficacy of RTI and PI (ξ) increases, the **A.** time of transition and **B.** viral load at the time of transition is reduced (see equation S.15).

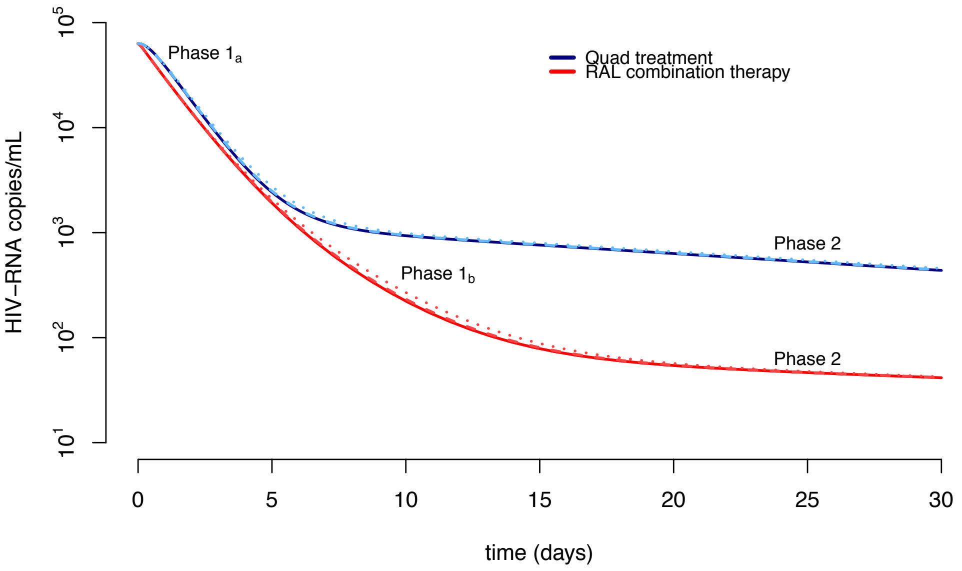

Figure F. Viral Load decay for different values of η and ε. Solid lines, dashed lines and dotted lines present the cases of the model using estimates for the best fits of the model in equation S.13 to the data, but for the cases η=ε=0.95, η=ε=0.9 and η=ε=0.8, respectively. There was not significant difference in the viral load profiles.

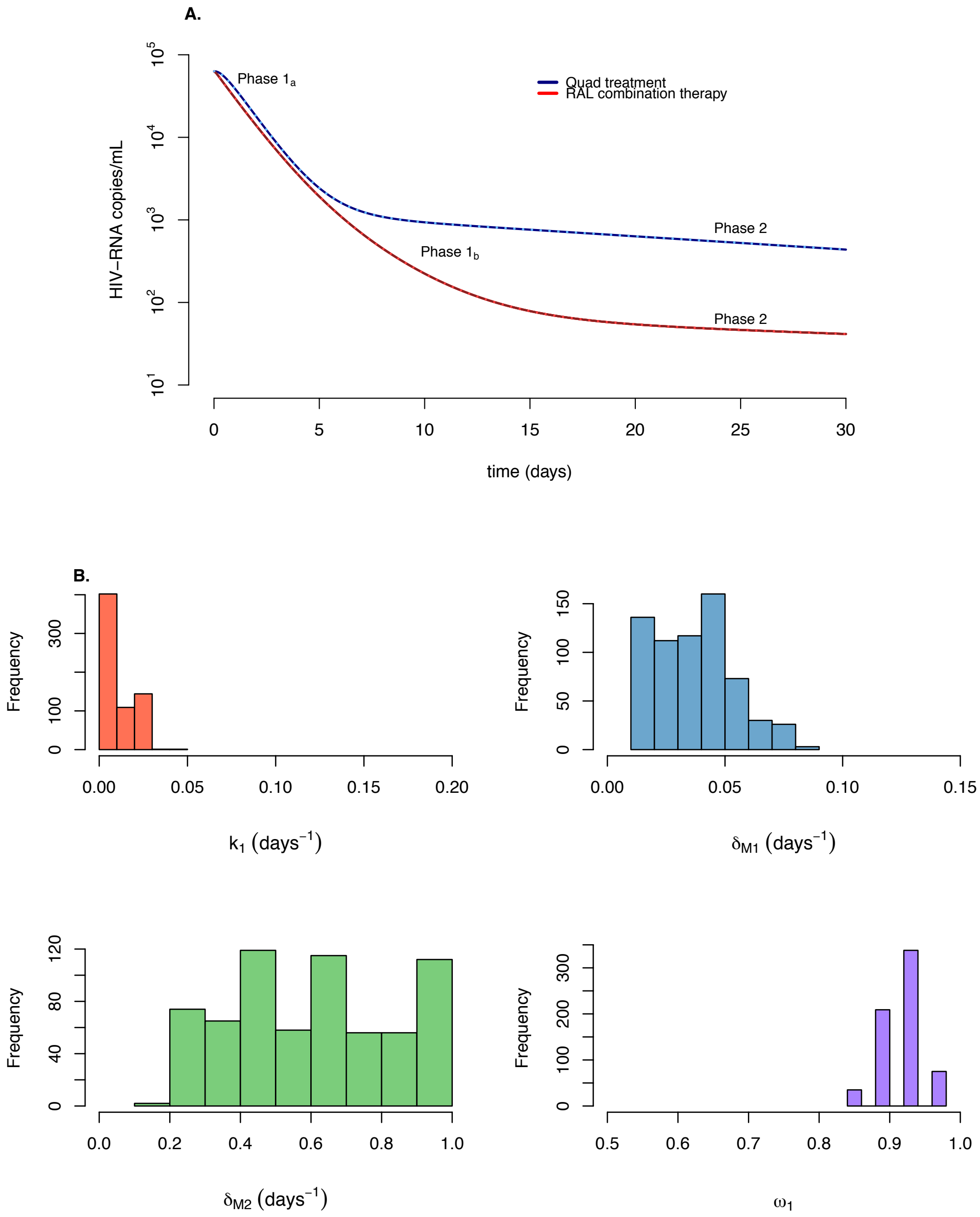

Figure G. A. Viral load decline using best fits presented in section 4.4 but using the model in equation S.17, assuming η=0.95. Solid red and blue lines present simulations for RAL-containing and RAL-free regimens, assuming η_1=0.95. Dashed light red and light blue present the same respective simulations but assuming η_1=0.9 and 0.8. No significant differences are observed by changing η_1. **B.** Simulations using the same criteria and assumptions presented in Fig. A, but using the model in equation S.17 and narrowing the set of values for the reduction of the second phase by raltegravir between 85-95%, fixing ω=0.94 and changing the value of ω_1 from 0.5 to 1. The figure presents that to have curves similar to the data even by changing ω_1, the values of δ_{M2} still has to be greater than δ_{M1}+k_1 as obtained by our analysis.

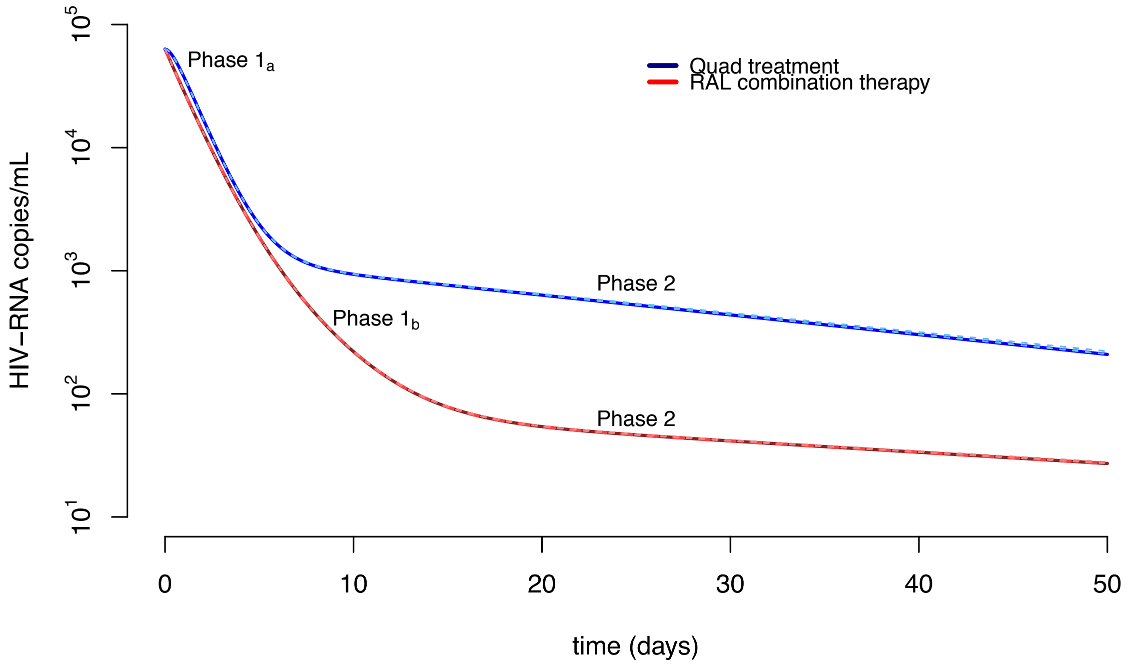

Figure H. Viral load decline assuming that target cells are not constant. Solid lines present the predicted viral load decline from the best fit parameters in model S.13 (constant target cells) to the data for each treatment case: RAL-free (blue) or RAL-containing regimens (red). Dashed light-blue and light-red lines present the 48 cases simulating equations S.19 (variable target cells) choosing values for d equal to 0.1, 0.01 and 0.001 day^{-1}, and values for β and β_2 equal to 10^{-8}, 10^{-7}, 10^{-6} and 10^{-5} ml day^{-1}. There is no significant difference in the viral load profiles by assuming constant or variable target cells during the initial 50 days of treatment.

6 Supplementary Tables

Table A. Population parameter estimates of fitting the model in equation (S.3) to the three sets of data simultaneously. The following parameters were fixed: $f_I = 0.98$, k=2.6 day^{-1} and c=23 day^{-1}. For RAL+RTI we used η=0.95, ε=0 and ω=0.94; for RTI+PI, η=ε=0.95 and ω=0.

Estimated Parameters						-2log(L)	AIC	ΔAIC
k_1 [day^{-1}]	δ_1 [day^{-1}]	δ_2 [day^{-1}]	$\log_{10} V_0$ [copies ml^{-1}]	δ_{M1} [day^{-1}]	δ_{M2} [day^{-1}]			
0.023 (0.02)	0.25 (0.04)	0.85 (0.04)	4.81 (0.07)	0.01 (0.006)	0.99 (1.4)	29.16	53.16	0
0.02 (0.01)	0.99 (3e-5)	0.83 (0.04)	4.80 (0.07)	0.02 (0.01)	0.22 (0.03)	48.46	72.46	19.3

Table B. Population parameter estimates of fitting the model in equation (S.3) to the three sets of data simultaneously, assuming δ_{M1} fixed. The following parameters were fixed f_I = 0.98, k=2.6 day^{-1} and c=23 day^{-1}. For RAL+RTI we used η=0.95, ε=0 and ω=0.94; for RTI+PI, η=ε=0.95 and ω=0.

Estimated Parameters					Fixed Parameters	-2log(L)	AIC	ΔAIC
k_1 [day^{-1}]	δ_1 [day^{-1}]	δ_2 [day^{-1}]	$\log_{10} V_0$ [copies ml^{-1}]	δ_{M2} [day^{-1}]				
6e-5 (7e-5)	1.46 (1.4)	0.84 (0.04)	4.81 (0.07)	0.2 (0.03)	δ_{M1}=0.1	63.98	85.98	36.64
0.02 (0.03)	0.99 (NaN)	0.83 (0.03)	4.79 (0.07)	0.23 (0.03)	δ_{M1}=0.05	50.82	72.82	23.48
0.01 (0.01)	0.23 (0.04)	0.86 (0.04)	4.81 (0.07)	0.98 (1.4)	δ_{M1}=0.02	28.45	50.45	1.11
0.038 (0.02)	0.24 (0.04)	0.86 (0.04)	4.80 (0.07)	0.90 (1.1)	δ_{M1}=0.01	27.34	49.34	0
0.05 (0.02)	0.27 (0.04)	0.85 (0.04)	4.80 (0.07)	0.99 (1.4)	δ_{M1}=0.005	35.48	57.48	8.14

Table C. Population parameter estimates of fitting the model in equation (S.3) to the three sets of data simultaneously, assuming δ_{M1} fixed and $\delta_{M2}=\delta_2$ (rewriten in equation S.13). The following parameters were fixed $f_I = 0.98$, k=2.6 day^{-1} and c=23 day^{-1}. For RAL+RTI we used η=0.95, ε=0 and ω=0.94; for RTI+PI, $\eta=\varepsilon$=0.95 and ω=0.

**Estimated Parameters**					**Fixed Parameters**	**-2log(L)**	**AIC**	**ΔAIC**
**k_1 [day^{-1}]**	**δ_1 [day^{-1}]**	**δ_2 [day^{-1}]**	**$\log_{10} V_0$ [copies ml^{-1}]**	**δ_{M1} [day^{-1}]**				
5e-5 (6e-5)	0.23 (0.04)	0.85 (0.03)	4.8 (0.07)	0.0213 (0.007)		24.75	46.75	2.47
0.005 (0.007)	0.23 (0.04)	0.86 (0.04)	4.81 (0.07)	-	δ_{M1}=0.05	73.76	91.76	47.48
0.005 (0.007)	0.23 (0.04)	0.86 (0.03)	4.81 (0.07)	-	δ_{M1}=0.03	36.97	54.97	10.69
0.017 (0.01)	0.23 (0.04)	0.85 (0.03)	4.80 (0.07)	-	δ_{M1}=0.02	26.28	44.28	0
0.037 (0.02)	0.24 (0.04)	0.85 (0.03)	4.80 (0.07)	-	δ_{M1}=0.01	26.87	44.87	0.59
0.048 (0.02)	0.25 (0.04)	0.84 (0.03)	4.80 (0.07)	-	δ_{M1}=0.005	28.23	46.23	1.95

Table D. Population parameter estimates of fitting the model in equation (S.3) to the three sets of data simultaneously, assuming δ_{M1} fixed, δ_{M2}=δ_2 and 100% efficacy for RTIs and PIs (with solution shown in equation S.16). The following parameters were fixed f_I = 0.98, k=2.6 day^{-1} and c=23 day^{-1}. For RAL+RTI we used η=1, ε=0 and ω=0.94; for RTI+PI, η=ε=1 and ω=0.

**Estimated Parameters**					**Fixed Parameters**	**-2log(L)**	**AIC**	**ΔAIC**
**k_1 [day^{-1}]**	**δ_1 [day^{-1}]**	**δ_2 [day^{-1}]**	**$\log_{10} V_0$ [copies ml^{-1}]**	**δ_{M1} [day^{-1}]**				
0.06 (0.04)	0.22 (0.04)	0.84 (0.03)	4.8 (0.07)	0.004 (0.008)		26.74	46.74	3.54
0.0004 (0.0008)	0.19 (0.04)	0.86 (0.03)	4.81 (0.07)	-	δ_{M1}=0.05	72.39	90.39	47.19
0.018 (0.01)	0.22 (0.05)	0.85 (0.03)	4.81 (0.07)	-	δ_{M1}=0.03	39.68	57.68	14.48
0.014 (0.01)	0.22 (0.04)	0.85 (0.03)	4.80 (0.07)	-	δ_{M1}=0.02	25.20	43.20	0
0.040 (0.02)	0.21 (0.04)	0.85 (0.03)	4.80 (0.07)	-	δ_{M1}=0.01	25.53	43.53	0.33
0.043 (0.02)	0.23 (0.04)	0.84 (0.03)	4.80 (0.07)		δ_{M1}=0.005	28.00	46.00	2.8

Table E. Individual parameter estimates. Rows with patient IDs from P202 to P212 present the best individual estimates for individuals under quad-regimen. Rows with patient IDs from Pat1 to Pat32 present the best individual estimates for individuals under RAL-combination therapy. Rows with patient IDs from M1 to M100 present the best individual estimates for individuals under RAL-monotherapy. All fits assumed $\delta_2 = \delta_{M2}$ and δ_{M1}=0.02 day^{-1} (equation S.3, rewritten in S.13).

Patient-ID	Mode				Mean				STD			
	$\log_{10} V_0$	δ_1	k_1	δ_2	$\log_{10} V_0$	δ_1	k_1	δ_2	$\log_{10} V_0$	δ_1	k_1	δ_2
**P202**	4.98	0.23	0.052	0.79	4.98	0.35	0.05	0.81	0.07	0.34	0.02	0.1
**P204**	4.4	0.22	0.052	1.14	4.39	0.27	0.045	1.13	0.07	0.2	0.02	0.11
**P205**	5.83	0.23	0.034	0.99	5.8	0.3	0.024	0.96	0.08	0.26	0.02	0.13
**P206**	5.99	0.26	0.092	0.93	5.96	0.34	0.085	0.9	0.07	0.31	0.01	0.12
**P207**	4.66	0.25	0.003	1	4.66	0.34	0.004	1.01	0.06	0.46	0	0.12
**P210**	5.31	0.24	0.005	0.63	5.33	0.38	0.006	0.67	0.08	0.36	0.01	0.11
**P211**	4.91	0.21	0.114	0.94	4.88	0.28	0.107	0.91	0.07	0.26	0.02	0.1
**P212**	4.46	0.28	0.006	0.93	4.46	0.47	0.006	0.92	0.07	0.79	0.01	0.15
**Pat1**	4.92	0.11	0.012	0.75	4.93	0.11	0.021	0.77	0.06	0.04	0.03	0.1
**Pat2**	4.5	0.15	0.017	0.8	4.51	0.15	0.076	0.81	0.06	0.04	0.13	0.1
**Pat5**	4.73	0.18	0.013	1.2	4.73	0.19	0.031	1.21	0.07	0.05	0.06	0.16
**Pat6**	6.02	0.34	1.498	0.96	6.01	0.45	1.41	0.92	0.07	0.33	0.27	0.12
**Pat13**	4.72	0.25	0.019	0.64	4.72	0.33	0.053	0.63	0.06	0.22	0.09	0.08
**Pat15**	5.07	0.11	0.012	0.88	5.07	0.11	0.027	0.88	0.07	0.04	0.04	0.12
**Pat16**	4.5	0.11	0.022	0.78	4.5	0.11	0.074	0.78	0.06	0.04	0.12	0.1
**Pat26**	4.57	0.12	0.015	0.86	4.56	0.12	0.048	0.86	0.07	0.04	0.08	0.12
**Pat27**	4.51	0.2	0.017	0.86	4.53	0.22	0.127	0.89	0.07	0.06	0.32	0.12
**Pat28**	4.89	0.37	0.017	0.84	4.87	0.54	0.03	0.81	0.06	0.41	0.05	0.09
**Pat32**	5.05	0.18	0.016	0.75	5.03	0.21	0.074	0.72	0.07	0.12	0.22	0.11
**M1**	4.01	0.31	0.017	1.03	3.99	0.46	0.1	1.01	0.09	0.4	0.36	0.12
**M2**	4.43	0.61	0.017	0.78	4.41	0.8	0.084	0.77	0.08	0.43	0.21	0.07
**M4**	3.9	0.3	0.017	0.83	3.89	0.42	0.097	0.82	0.09	0.39	0.27	0.11
**M6**	4.32	0.31	0.017	0.95	4.31	0.43	0.154	0.95	0.09	0.44	0.7	0.11
**M7**	4.78	0.15	0.017	0.73	4.78	0.17	0.113	0.74	0.1	0.14	0.36	0.1
**M8**	4.39	0.18	0.017	0.66	4.39	0.25	0.082	0.67	0.09	0.27	0.2	0.08
**M10**	4.4	0.74	0.017	1.13	4.39	1.02	0.074	1.13	0.09	0.67	0.18	0.1
**M11**	3.73	0.22	0.017	0.88	3.73	0.33	0.127	0.85	0.1	0.43	0.44	0.13

**M12**	3.94	0.39	0.017	0.98	3.93	0.55	0.114	0.99	0.09	0.37	0.47	0.11
**M13**	5.06	0.22	0.017	0.74	5.04	0.25	0.167	0.73	0.08	0.18	0.75	0.09
**M15**	4.14	0.23	0.017	0.97	4.13	0.32	0.069	0.96	0.09	0.3	0.12	0.12
**M16**	4.84	0.59	0.017	1.08	4.84	0.81	0.078	1.07	0.09	0.4	0.19	0.1
**M17**	4.59	0.1	0.017	1.3	4.57	0.1	0.097	1.28	0.09	0.05	0.28	0.13
**M18**	4.01	0.29	0.017	1.02	4.01	0.46	0.081	1.02	0.09	0.39	0.19	0.12
**M84**	5.49	0.18	0.017	0.76	5.47	0.2	0.122	0.75	0.08	0.14	0.55	0.09
**M85**	5.24	0.39	0.017	0.98	5.24	0.5	0.22	0.98	0.09	0.31	0.71	0.12
**M86**	5.21	0.34	0.017	0.98	5.19	0.44	0.103	0.94	0.09	0.25	0.28	0.12
**M88**	5.2	0.3	0.017	0.7	5.19	0.43	0.095	0.69	0.1	0.31	0.31	0.08
**M89**	5.15	0.09	0.017	0.58	5.13	0.15	0.096	0.56	0.08	0.18	0.36	0.08
**M90**	4.94	0.11	0.017	0.54	4.95	0.16	0.174	0.55	0.08	0.16	0.60644	0.083699
**M91**	5.14	0.33	0.017	0.66	5.14	0.36	0.089	0.67	0.08	0.19	0.22589	0.075304
**M92**	5.11	0.12	0.017	0.65	5.12	0.13	0.13	0.66	0.1	0.1	0.46749	0.097156
**M93**	5.34	0.21	0.017	1.1	5.32	0.2	0.12	1.09	0.1	0.06	0.48711	0.12504
**M94**	5.18	1.14	0.017	1.2	5.19	1.43	0.068	1.21	0.08	0.58	0.16883	0.084751
**M96**	5.03	0.25	0.017	0.7	5.03	0.34	0.087	0.71	0.08	0.56	0.29619	0.087587
**M97**	5.05	0.11	0.017	0.92	5.03	0.11	0.093	0.89	0.09	0.05	0.29416	0.12036
**M99**	5.78	0.13	0.017	0.87	5.77	0.13	0.087	0.86	0.09	0.06	0.26825	0.10572
**M100**	5.14	0.26	0.017	0.92	5.13	0.27	0.078	0.92	0.09	0.1	0.17597	0.10756

7 Supplementary Movie Legend

Movie S1. Animation of the dynamics predicted by the model S.13 for the best fits to the RAL+RTI (red) and QUAD therapy (blue) data. The panels at the top show in block diagrams the decay of the pre-integration cell compartments I_1 and M_1, and the productively infected cells compartment I_2 in both, RAL combination therapy (red) and QUAD therapy (blue). Horizontal arrows indicate the start of the second phase for each therapy in each compartment. The bottom panel shows the corresponding decay in ($\log_{10}$) viral load over time, where the vertical dashed lines indicate the start of the second phase (QUAD therapy in blue and RAL-combination therapy in red).

Virus is produced by productively infected cells (I_2). Initially they are lost quickly by death (rate d_2), for both types of treatment. At the same time these cells (I_2) are replenished by infected cells progressing through integration (I_1 and M_1). In the presence of RTIs and PIs without InSTIs, the pool of cells with fast integration (I_1) decays quickly and the second phase starts when the main contribution to I_2 comes from the conversion of slowly integrating cells (M_1) at rate k_1. The productively infected cells in the QUAD panel then have an effective decay rate given by ($\sim\delta_{M1}+k_1$), which is the rate limiting step. In the presence of an InSTI, the pool of cells that can integrate fast (I_1) decreases more slowly, because integration is slowed down but not prevented completely. This results in phase 1b with slope ($\sim\delta_1+k(1\text{-}\omega)$) equal to the slower decay of cells in I_1. Together phases 1a and 1b last longer than phase 1, because it takes longer to lose cells in I_1. The second phase for RAL combination therapy starts when the main contribution to productively infected cells comes from M_1, which in the presence of an InSTI occurs later, because integration is slowed down in this compartment too. This explains the lower viral load level at the start of the second phase with an InSTI regimen.

Fixed parameter values are: $V_I(0)/V(0) = 0.98$,δ_{M1}=0.02 day^{-1}, k=2.6 day^{-1} and c=23 day^{-1} based on previous studies [1,7,8]. In addition, for RAL combination we used η=0.95, ε=0 and ω=0.94, for the quad therapy $\eta=\varepsilon$=0.95 and ω=0, and for RAL monotherapy $\eta=\varepsilon$=0 and ω=0.997 [1]. The estimated best-fit population parameters are (estimated standard deviation in parenthesis): δ_1=0.23 (0.04) day^{-1}, k_1=0.017 (0.01) day^{-1}, $\delta_{M2}=\delta_2$=0.85 (0.07) day^{-1} and $V(0)$ = 4.8 (0.07).

8 References

1. Andrade, A. *et al.* Early HIV RNA decay during raltegravir-containing regimens exhibits two distinct subphases (1a and 1b). *AIDS Lond. Engl.* **29,** 2419–2426 (2015).

2. Perelson, A. S., Neumann, A. U., Markowitz, M., Leonard, J. M. & Ho, D. D. HIV-1 dynamics in vivo: virion clearance rate, infected cell life-span, and viral generation time. *Science* **271,** 1582–1586 (1996).

3. Perelson, A. S. *et al.* Decay characteristics of HIV-1-infected compartments during combination therapy. *Nature* **387,** 188–191 (1997).

4. Sedaghat, A. R., Dinoso, J. B., Shen, L., Wilke, C. O. & Siliciano, R. F. Decay dynamics of HIV-1 depend on the inhibited stages of the viral life cycle. *Proc. Natl. Acad. Sci.* **105,** 4832–4837 (2008).

5. Wang, X., Song, X., Tang, S. & Rong, L. Dynamics of an HIV model with multiple infection stages and treatment with different drug classes. *Bull. Math. Biol.* 1–28 (2016). doi:10.1007/s11538-016-0145-5

6. Sedaghat, A. R., Siliciano, R. F. & Wilke, C. O. Constraints on the dominant mechanism for HIV viral dynamics in patients on raltegravir. *Antivir. Ther.* **14,** 263–271 (2009).

7. Markowitz, M. *et al.* A novel antiviral intervention results in more accurate assessment of human immunodeficiency virus type 1 replication dynamics and T-cell decay in vivo. *J. Virol.* **77,** 5037–5038 (2003).

8. Ramratnam, B. *et al.* Rapid production and clearance of HIV-1 and hepatitis C virus assessed by large volume plasma apheresis. *The Lancet* **354,** 1782–1785 (1999).
